# East Timor as an important source of cashew (*Anacardium occidentale* L.) genetic diversity

**DOI:** 10.7717/peerj.14894

**Published:** 2023-04-24

**Authors:** Lara Guterres, João Barnabé, André Barros, Alberto Bento Charrua, Maria Cristina Duarte, Maria M. Romeiras, Filipa Monteiro

**Affiliations:** 1 Nova School of Business and Economics, Campus de Carcavelos, Universidade Nova de Lisboa, Cascais, Portugal; 2 LEAF—Linking Landscape, Environment, Agriculture and Food Research Center, Associated Laboratory TERRA, School of Agriculture (ISA), University of Lisbon, Lisbon, Portugal; 3 Universidade Nacional Timor Lorosa’e (UNTL), Díli, East-Timor; 4 cE3c—Centre for Ecology, Evolution and Environmental Changes & CHANGE—Global Change and Sustainability Institute, Faculty of Sciences, University of Lisbon, Lisbon, Portugal; 5 Innate Immunity and Inflammation Laboratory, Instituto Gulbenkian de Ciência (IGC), Oeiras, Portugal; 6 Department of Earth Sciences and Environment, Faculty of Science and Technology, Licungo University, Beira, Mozambique

**Keywords:** Genetic diversity, SSRs, Population structuring, Southeast Asia, Diversity hotspots

## Abstract

**Background:**

Cashew (*Anacardium occidentale* L.) is a crop currently grown in several tropical countries because of the economic importance of cashew nuts. Despite its enormous economic worth, limited research has been conducted on the molecular diversity of cashew genetic resources. In this study, a wide comprehensive assessment of the genetic diversity of cashew trees in East Timor was performed using microsatellites (SSRs) to evaluate intraspecific diversity and population structuring.

**Methods:**

A total of 207 individual cashew trees, including trees from East Timor (11), and outgroup populations from Indonesia (one) and Mozambique (two), were analyzed with 16 cashew-specific SSRs. A comprehensive sampling of cashew trees within East Timor was performed, covering the distribution of cashew orchards in the country. Genetic diversity indices were calculated, and population structuring was determined using three different approaches: genetic distances (UPGMA and NJ), AMOVA, and individual-based clustering methods through Bayesian (STRUCTURE) and multivariate (DAPC) analyses.

**Results:**

The population structuring analysis revealed that the genetic diversity of cashew populations in East Timor was higher in this study than previously reported for cashew trees. A higher allelic richness was found within cashew populations in East Timor compared with the outgroup populations (Mozambique and Indonesia), reinforced by the presence of private alleles. Moreover, our study showed that cashew populations in East Timor are grouped into two dissimilar genetic groups, which may suggest multiple cashew introductions over time. These new cashew genetic resources could be explored for future crop improvement.

**Conclusions:**

Crop diversity underpins the productivity, resilience, and adaptive capacity of agriculture. Therefore, this study provides useful information regarding genetic diversity and population structure that can be harnessed to improve cashew production in East Timor. This data is also important to creating a country-specific genetic cashew signature to increase cashew market value.

## Introduction

Cashew (*Anacardium occidentale* L., Anacardiaceae) is a tropical evergreen tree that can thrive in both dry and wet tropical climates. Many tropical countries use various parts of the cashew tree for consumption as well as for medicinal and industrial purposes (Salehi et al., 2019). Among the various parts of the plant, the cashew kernel has the highest market value and is an export-oriented commodity, also known as a cash crop, in several tropical countries (Monteiro et al., 2015; Monteiro et al., 2017). The highest cashew-producing countries, mainly in West Africa (Guinea-Bissau and Côte d’Ivoire) and Southeast Asia (India and Vietnam), focus primarily on exporting the nuts, which are the primary source of income derived from cashew trees for both governments and farmers (Havik et al., 2018). Over the past two decades, a rising worldwide consumption of cashew nuts has led to increased global market demand. Cashews accounted for 17% of world tree nut production in 2019/20, making it the third most popular tree nut after almonds and walnuts (International Nut and Dried Fruit Council Foundation, 2020). The recent popularity of cashews is mainly attributed to a trend towards healthier food habits. As a rich source of plant-based protein and dietary minerals, and because of their low-fat content, cashews have become one of the most produced and valued tree nuts worldwide, together with almonds, pistachios, and walnuts (Pradhan, Peter & Dileep, 2020).

Despite the enormous economic importance of cashew trees, studies evaluating the molecular diversity of cashew genetic resources have been scarce. Most studies on this topic were performed in the top cashew-producing countries and with different molecular markers, namely microsatellites (SSRs, simple sequence repeats) and RAPDs (random amplified polymorphic DNA). For example, the genetic diversity of cashew accessions have been characterized in Côte d’Ivoire (Kouakou et al., 2020), Nigeria (Aliyu & Awopetu, 2007), India (Archak et al., 2009), Tanzania (Mneney, Mantell & Bennett, 2001), Malawi (Chipojola et al., 2009), and Brazil (Dos Santos et al., 2019). Overall, these studies highlight the narrow genetic diversity of cashew accessions when compared to Brazil, the native country of origin of cashew trees, where a higher diversity was observed.

Cashew trees were one of the many tropical crops introduced by the Portuguese from Brazil into the African continent in the sixteenth century as part of the Columbian Exchange Event (Havik et al., 2018). In Asia, cashew trees were most likely introduced through Goa (India), Portugal’s main settlement in the East Indies in the sixteenth century (Massari, 1994). As a result of cashew trees adapting well to the soil conditions in India, cashew products were explored beyond the nut, such as the local fermented brew, *feni*, made from ripened cashew apples. After their introduction in India, cashew trees spread to South Asia on the Moluccas Islands, Indonesia (Nair, 2010) and thereafter to the present day countries of Vietnam, Philippines, Malaysia, Thailand, and Sri Lanka (Havik et al., 2018). Despite the different timelines of introduction, cashew trees were first established to help combat deforestation in both African and Asian countries (Monteiro et al., 2017; Havik et al., 2018). However, these two tropical regions have turned cashew into one of the most exported agriculture commodities, though this happened at different paces in Africa and Asia. While most African countries (except for Mozambique, Nigeria, and Côte d’Ivoire) are amongst the highest exporters of unprocessed cashew nuts, Asia’s cashew industry also processes the imported raw nuts from these African countries (*e.g.*, Guinea-Bissau, Senegal, Guinea, and Burkina Faso).

East Timor is a small island country located in Asia, bordered by Indonesia (Government of East Timor, 2015). Agriculture is the most important economic sector providing subsistence to an estimated 80% of the population (Harmadi & Gomes, 2013). Of the 225,000 ha of cultivated land area in the country, 165,000 ha is arable land (with different annual crops) and 60,000 ha is devoted to permanent crops such as rice, corn, cassava, coffee, coconut, and other industrial crops (MAFF, 2014). Coffee, maize, and rice are the most important staple crops in the area (MAFF, 2014; Borges, 2018). In East Timor, the cashew has been developed as an industrial crop since the 1990s, with over 3,200 ha planted on 6,500 small farms. The armed period with Indonesia resulted in several cashew orchards being burnt. In 2008, about 125,000 cashew trees (about 800 ha) remained, growing in ten districts (Bobonaro, Manatuto, Oecusse, Cova Lima, Ainaro, Manufahi, Viqueque, Baucau, Lospalos, and Dili), with a relatively low average yield per ha (270–300 kg/ha, MAFF, 2014; Peng et al., 2015). Cashew varieties planted include the locally recognized variety from Indonesia, and more recently, varieties from Brazil and Australia (Peng et al., 2015), to increase yield per ha. As part of the current East Timor Strategic Development Plan 2011-2030 (República Democrática de Timor-Leste, 2011), cashew is being used as an export commodity to help increase the country’s agriculture remittance.

Studies on the cashew germplasm have mostly been done in major cashew-producing countries (*e.g.*, Côte d’Ivoire (Kouakou et al., 2020), Nigeria (Aliyu & Awopetu, 2007), India (Archak et al., 2009), Tanzania (Mneney, Mantell & Bennett, 2001)) and a narrow genetic diversity has been observed. However, in countries more recent to commercial cashew production with low or no presence in international cashew markets, the genetic diversity of cashew trees remains to be discovered. Thus, evaluating the genetic resources of emerging cashew-producing countries, such as East Timor, is key for assessing on farm diversity as well as the overall current genetic diversity of cashew trees in the country. These results will help identify the potential of untapped genetic resources for future cashew crop improvement. This study aims to assess the genetic diversity of the cashew populations cultivated in East Timor, an emergent cashew-producing country, using highly informative molecular markers as microsatellites, which are effective in evaluating intra-specific genetic diversity and population structuring. To accomplish this objective, 11 cashew orchards from East Timor were assessed with 16 cashew-specific microsatellites (Croxford, Robson & Wilkinson, 2006) applied to screen in-country genetic diversity. To assess the genetic signature of East Timor cashew trees, an Indonesian cashew population was included as an outgroup along with two populations from Mozambique working as a continental outgroup. Our work is pioneering new research, as no studies have been conducted in East Timor using a highly comprehensive sampling scheme (over 11 populations) in an emerging cash crop, such as cashew.

## Materials & Methods

### Plant sample collection and site

**Figure 1:**
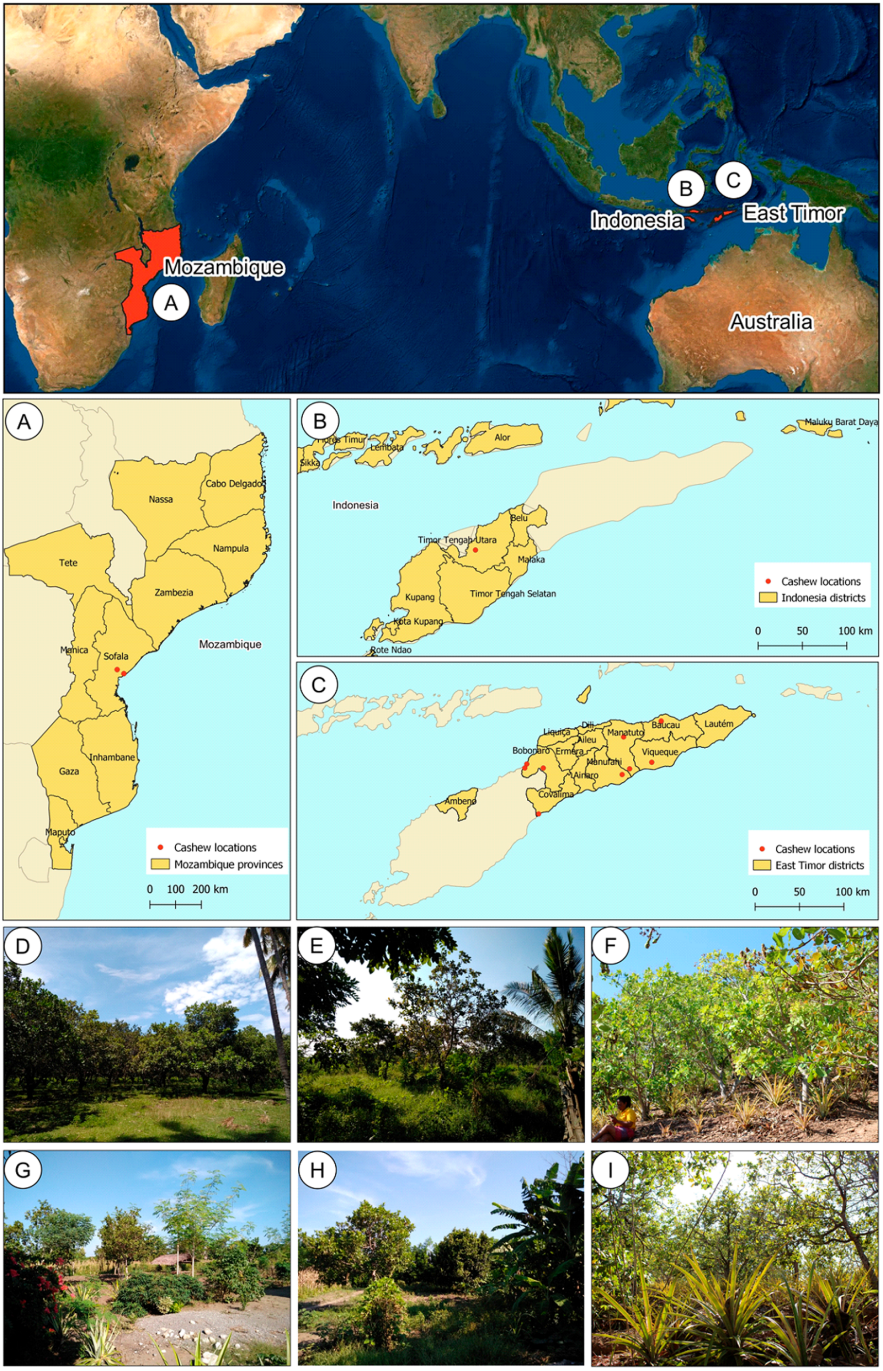
Geographical location of cashew populations studied in Mozambique (A), Indonesia, (B) and East Timor (C), and illustrative orchards from East Timor (D–H) and Indonesia (I). Illustrative orchards from some plantations sampled in East Timor (D–H): Natarbora in the Manatuto district (D), Mailiana in the Bobonaro district (E), Triloca in the Baucau district (F), Fatucahi in the Manufahi district (G), Viqueque district (H), and Kefamenanu in Indonesia (I).

East Timor is divided into six different agro-ecological zones (ARPAPET, 1996) that shape the diverse ecological and agricultural landscapes in the country, where the north coast is far drier than the south (ARPAPET, 1996; Fox, 2003). Cashew populations were sampled from different orchards in East Timor (11), Indonesia (one), and Mozambique (two) (Figs. 1A–1C). Each population is represented by 12-18 individual cashew trees from an orchard (Table 1), with a total of 207 samples analyzed. Leaves were sampled and preserved in silica gel until further processing. Within East Timor (Fig. 1C), the populations of 11 cashew orchards were sampled, covering six districts and cashew-producing regions (Figs. 1D–1I). The studied districts included four of the six agro-ecological zones in the country, namely: the Northern Coast Lowlands: Manatuto (Kribas); Northern Slopes: Baucau; Northern Uplands: Bobonaro; and South Coast Lowlands: Manatuto (Natarbora), Cova Lima, Manufahi, and Viqueque. A population from Kefamenanu, Indonesia was also included (Fig. 1B); this is a district in Kota Kefamenanu and is located in the North Central Timor Regency which borders East Timor’s Oecusse enclave. It is one of the few Indonesian regions that has a land border with another country; therefore, this population is essential for identifying a possible genetic connection between the cashew trees in East Timor and those from the only major region of Indonesia with a strong land connection with East Timor. Two cashew populations from Mozambique (Fig. 1A) were also included, working as a possible population outgroup between continental and island regions. This cashew population is also important because, due to the strong historical connection with other Portuguese colonies during the 16th and 17th centuries, Mozambique was an important continental outpost for the exploration of the Southeast Asian islands (De Carvalho & Mendes, 2016; Havik et al., 2018). All DNA samples are stored at the Instituto Superior de Agronomia, University of Lisboa (Portugal) and are available upon request.

**Table 1:** Populations by country, district, and location with geographical coordinates and total individuals sampled (N).

Country	District	Location	Population	Latitude	Longitude	N
East Timor (ET)	Manatuto	Kribas	ETK	−8.650	125.983	17
			Natarbora	ETNA	−8.974	126.045	14
		Baucau	Triloca	ETTR1	−8.490	126.370	14
						ETTR2	−8.491	126.370	14
				ETTR3	−8.487	126.370	16
		Cova Lima	Suai	ETSU	−9.432	125.108	16
		Bobonaro	Sanirin	ETSAN	−8.925	124.986	18
				Maliana	ETMA	−8.966	125.156	14
			Batugade	ETBAT	−8.965	124.966	13
		Manufahi	Fatucahi	ETFA	−9.032	125.968	14
	Viqueque	Viqueque	ETV	−8.907	126.273	14
Indonesia (IND)	Kota Kefamenanu	Kefamenanu	IND	−9.437	124.485	16
Mozambique (MZ)	Sofala	Beira	MZB	−19.723	34.982	12
		Dondo	MZD	−19.571	34.715	15
Total						**207**

### Genomic DNA extraction

Individual tender leaves collected from each cashew population were used for genomic DNA (gDNA) analysis, with gDNA extracted with the InnuPREP Plant DNA Kit (Analytik Jena, Germany) following the manufacturer’s instructions with minor modifications. Approximately 100 mg of each leaf collected in the field were ground with a mortar and a pestle in liquid nitrogen, with about 25 mg of the roughly ground leaves used for the subsequent gDNA extraction protocol, by adding 400 µL of OPT lysis solution and grinding the biological material with an Eppendorf-pestle in a 1.5 mL tube. An initial incubation was done for 1 h at 65 °C, followed by the addition of 100 µL of precipitation buffer and a 5-min incubation at room temperature; the supernatant was then recovered by centrifugation at a maximum speed of 11.000 × g for 5 mins. The supernatant was then transferred to a pre-filter receiver and centrifuged at 11.000 × g for 1 min. Subsequently, 4 µL of RNAse A solution (100 mg/mL) was added and samples were incubated for 30 mins at 37 °C. After RNAse treatment, 200 µl of SBS binding solution was added and then centrifuged at 11.000 × g for 2 mins. Two washing steps with 650 µL of MS washing solution were then performed on the recovered supernatant, which was then centrifuged at 11.000 × g for 1 min. The gDNA was eluted in 40 µL of AE buffer, left to incubate at room temperature for 15 mins, and recovered by centrifugation at 11,000 × g for 1 min. DNA purity and concentration were measured at 260/280 *η*m and 260/230 *η*m using a spectrophotometer (NanoDrop-1000, Thermo Scientific, Waltham, MA, USA), while DNA integrity was verified by agarose gel electrophoresis at 0.8% in 1x TAE running Buffer (Merck, Darmstadt, Germany) for 30 mins at 90 volts and then visualized in a GelDoc XR image system (BioRad, Hercules, CA, USA).

### Microsatellite genotyping

A set of 16 available cashew-specific microsatellite (SSRs) markers (Croxford, Robson & Wilkinson, 2006) were selected for screening the genetic diversity of the populations, following three major criteria: (i) markers with a Polymorphism Information Content (PIC) value higher than 0.5, as a reference value to be considered as an informative marker, (ii) markers with high allelic diversity, (iii) and dinucleotide repeat markers to enable a clearer interpretation upon microsatellite genotyping, thus avoiding genotyping errors.

Before multiplexing, each SSR marker was validated in single-plex polymerase chain reactions (PCR) using a three-primer PCR approach (Schuelke, 2000) to assess both reaction reproducibility and/or the presence of PCR artifacts upon fragment analysis (Monteiro et al., 2016). Each SSR was PCR amplified in a 25 µL volume reaction following the cycling conditions previously described by Croxford, Robson & Wilkinson (2006), using a HotStar Taq DNA Polymerase kit (QIAGEN, Germany), as per the manufacturer’s instructions. Next, the amplified SSR fragments were run through an ABI 3130XL sequencer (Applied Biosystems, Waltham, MA, USA) using the internal size standard of the GS500 LIZ (Applied Biosystems, Waltham, MA, USA) at the STAB VIDA company (Costa da Caparica, Portugal). Allele calling was performed in GeneMapper v 3.7 (Applied Biosystems, Waltham, MA, USA). The success of the co-amplification loci was assessed using the Multiplex Manager software v1.2 (Holleley & Geerts, 2009). Four SSR panels were assembled in 4-plex PCR reactions (Multiplex A, B, C, and D; Table 2) using four universal forward fluorescent-labelled primers following the methods outlined by Culley et al. (2013). To increase genotyping accuracy, a “PIG-tail” sequence was added at the 5′ end of each of the reverse primers (Brownstein, Carpten & Smith, 1996). PCR multiplex amplifications were carried out using the QIAGEN Multiplex PCR kit (QIAGEN, Germany) following the manufacturer’s protocol. Each PCR reaction included 25 µL of total volume after adding: 5 µL of 5x Q-Solution, 12.5 µL of 2x QIAGEN Multiplex PCR Master Mix (which includes HotStarTaq DNA Polymerase, PCR Buffer with 6 mM MgCl_2_, and dNTP mix), 50–100 *η*g gDNA, 2.5 *ρ*mol of each forward and reverse primer, 0.15 *ρ*mol of the tailed fluorescent-labeled primers (D1–D4), and a variable volume of ddH_2_O until the total volume reached 25 µL. Reactions were done in 96 well-plates; on each plate, one sample was repeated per run as a positive control for allele scoring. Negative PCR controls were also included. Hot start PCR methodology was used, heating the samples at 95 °C for 15 min. This was followed by a touchdown cycling protocol adapted from Croxford, Robson & Wilkinson (2006) as follows: five cycles of denaturation at 95 °C for 45 s; primer annealing at 68 °C for 5 min with −2 °C/cycle; a sequence extension at 72 °C for 1 min; five cycles of denaturation at 95 °C for 45 s; primers annealing (58 °C for Multiplexes A, C and D and 60 °C for Multiplex B) for 2 min with −2 °C/cycle; an extension step for 1 min at 72 °C; 27 cycles at 95 °C for 45 s, 47 ° C for 75 s, and 72 °C for 1 min; followed by a final extension step at 72 °C for 10 min. Next, the multiplex PCR products were run in an ABI 3130XL sequencer for a fragment analysis, as described earlier, and SSR allele sizes were aligned with the internal size standard. To improve SSR data quality, allele callings were checked manually, and ambiguous results were set as “missing data”. The resulting genotypic matrix was used for genetic diversity and population structuring analyses. The genotypic data generated from this study is freely available at FigShare in Guterres, Barnabé & Monteiro (2022).

**Table 2:** Loci used to screen 14 cashew populations. Primers sequences, multiplexing scheme, amplicon size range (bp), and amplicon expected size (bp) are provided. Following Culley et al. (2013), D1 (6-FAM): M13 (-21), 5′-TGTAAAACGACGGCCAGT-3′; D2 (NED,): T7term, 5′-CTAGTTATTGCTCAGCGGT-3′; D3 (VIC): M13modA, 5′-TAGGAGTGCAGCAAGCAT-3′; D4 (PET): M13modB, 5′-CACTGCTTAGAGCGATGC-3′. Italicized sequence at each reverse primer (GTTTCTT) identifies the “PIG-tail”.

Locus	Repeat motif	Primers (5′-3′)	Tailed Primer	Size range (Expected Size)	Multiplex
mAoR6	(AT)_5_(GT)_12_	F: CAAAACTAGCCGGAATCTAGC	D2	118–186 (143)	**A**
		R: *GTTTCTT* CCCCATCAAACCCTTATGAC				
mAoR17	(GA)_24_	F: GCAATGTGCAGACATGGTTC	D1	122-184 (124)		
		R: *GTTTCTT* GGTTTCGCATGGAAGAAGAG				
mAoR7	(AT)_2_(GT)_5_AT(GT)_5_	F: AACCTTCACTCCTCTGAAGC	D4	158-198 (178)		
		R: *GTTTCTT* GTGAATCCAAAGCGTGTG				
mAoR48	(GAA)_6_(GA)_3_	F: CAGCGAGTGGCTTACGAAAT	D3	130-186 (177)		
		R: *GTTTCTT* GACCATGGGCTTGATACGTC			
mAoR3	(AC)_12_(AAAAT)_2_	F: CAGAACCGTCACTCCACTCC	D4	140-282 (231)	**B**
		R: *GTTTCTT* ATCCAGACGAAGAAGCGATG				
mAoR42	(CAT)_9_TAT(CTT)_7_	F: ACTGTCACGTCAATGGCATC	D2	160-232(204)		
		R: *GTTTCTT* GCGAAGGTCAAAGAGCAGTC				
mAoR52	(GT)_16_(TA)_2_	F: GCTATGACCCTTGGGAACTC	D1	142-244 (202)		
		R: *GTTTCTT* GTGACACAACCAAAACCACA				
mAoR11	(AT)_3_(AC)_16_	F: ATCCAACAGCCACAATCCTC	D3	142-248 (234)		
		R: *GTTTCTT* CTTACAGCCCCAAACTCTCG			
mAoR2	(CA)_10_(TA)_6_	F: GGCCATGGGAAACAACAA	D3	172-322 (366)	**C**
		R: *GTTTCTT* GGAAGGGCATTATGGGTAAG				
mAoR33	(CT)_18_(AT)_19_	F: CATCCTTTTGCCAATTAAAAACA	D4	322-404 (354)		
		R: *GTTTCTT* CACGTGTATTGTGCTCACTCG				
mAoR35	(AG)_14_	F: *T* CTTTCGTTCCAATGCTCCTC	D2	142-180 (165)		
		R: *GTTTCTT* CATGTGACAGTTCGGCTGTT				
mAoR47	(TAAA)_2_(TA)_7_(AAT)	F: AAGAGCTGCGACCAATGTTT	D1	166-272 (161)		
		R: *GTTTCTTCTT* CTTGAACTTGACACTTCATCCA			
mAoR12	(AC)_12_ATAC(AT)_4_	F: CACCAAGATTGTGCTCCTG	D2	322-362 (324)	**D**
		R: *GTTTCTT* AAACTACGTCCGGTCACACA				
mAoR16	(GT)_8_(TA)_17_(GT)_3_	F: GGAGAAAGCAGTGGAGTTGC	D1	245-335 (256)		
		R: *GTTTCTT* CAAGTGAGTCCTCTCACTCTCA				
mAoR29	(TG)_10_	F: GGAGAAGAAAAGTTAGGTTTGAC	D3	164-364 (316)		
		R: *GTTTCTT* CGTCTTCTTCCACATGCTTC				
mAoR41	(ACC)_7_(AC)_3_	F: GCTTAGCCGGCACGATATTA	D4	162-177 (151)		
		R: *GTTTCTT* AGCTCACCTCGTTTCGTTTC			

### Genetic diversity analysis

Genotyping errors were assessed using MICRO-CHECKER v2.2.3 (Van Oosterhout et al., 2004), and the null alleles frequency was estimated using the EM algorithm of Dempster, Laird & Rubin (1977), as implemented in FreeNA (https://www1.montpellier.inra.fr/CBGP/software/FreeNA/). These values were computed as described by Chapuis & Estoup (2007), with 10,000 bootstrap iterations, alternatively using and not using the Excluding Null Alleles (ENA) method, after assessing the null allele frequencies. The Polymorphic Information Content (PIC) and genetic diversity indices were calculated with the Microsatellite Toolkit v.3.1.1 (Park, 2001) and GenALEx 6.5 (Peakall & Smouse, 2006), respectively, as previous described (Monteiro et al., 2016). These included the total allele number and mean alleles per locus (Na), private alleles, inbreeding coefficient (fixation index, *F*), observed (H_O_), and expected (H_e_) heterozygosity. Deviations from the Hardy-Weinberg equilibrium (HWE) were assessed for each locus-population combination and linkage disequilibrium (LD) to determine the extent of distortion from the independent segregation of loci using GenePop v.4.5 (Rousset, 2008). Statistical significance for both the HWE and LD was tested by running a Monte Carlo Markov chain (MCMC), each consisting of 10,000 iterations, and *p*-values were corrected for multiple comparisons (*p* < 0.000298, (0.05/168)) by applying a sequential Bonferroni correction (Rice, 2013).

### Population structuring

Population structure was addressed using three approaches: (i) using genetic distances to estimate relationships between populations; (ii) hierarchical genetic analysis by AMOVA; and (iii) individual-based clustering with a Bayesian (STRUCTURE) and a multivariate (DAPC, discriminant analysis of principal components) analysis.

#### Estimating relationships using genetic distances

Relationships between populations were estimated according to the methods described by Monteiro et al. (2016), specifically, the chord genetic distances introduced by Cavalli-Sforza and Edwards (DC, Cavalli-Sforza & Edwards, 1967) using the INA method computed in FreeNA (DC^INA^), and Nei’s D distance (Nei, 1972) was calculated in GenALEx 6.5. The unweighted pair group method with arithmetic mean (UPGMA) and neighbor-joining (NJ) trees were produced using the *ape* v3.4. package (Paradis, Claude & Strimmer, 2004) for R v4.1.0 (R Core Team, 2021) based on 10,000 bootstrap values, assessed by a boot function from the *poppr* v2.1.0. package (Kamvar, Tabima & Grunwald, 2014). Trees were further edited in FigTree v1.4.2 (Rambaut, 2014). Genetic distance was used to measure the relationship among populations using two separate groupings: the first included the cashew populations from all three countries (East Timor, Indonesia, and Mozambique), and the second excluded the continental populations from Mozambique.

#### Analysis of molecular variance (AMOVA)

An analysis of molecular variance (AMOVA, Weir & Cockerham, 1984; Hill, 1996) was done using ARLEQUIN v3.5.1.3 (Excoffier & Lischer, 2010) to assess the hierarchical distribution of the genetic variation of the cashew populations analysed. Significance was assessed after 1,000 permutations. Two three-level AMOVAs were performed: one using the cashew populations in each of the three countries (MZ =2; IND =1; ET =11) as individual groups, and the second considering only the cashew populations in East Timor and Indonesia as groups. In each AMOVA, the total variance was partitioned into components to account for pairwise differences between groups (V_a_, (1) MZ *vs* ET *vs* IND; (2) ET *vs* IND populations), differences among populations within those groups (V_b_), and differences among individuals within the populations (V_c_). Variance components (V_a_, V_b_, and V_c_) were used to calculate the fixation indices (*F*-statistics; *F*_CT_, *F*_SC_, *F*_ST_) according to Weir & Cockerham (1984).

#### Individual-based clustering analyses

Genetically distinct clusters were identified using two different methodologies, as previously described in Monteiro et al. (2016): a Bayesian clustering analysis using STRUCTURE (Pritchard, Stephens & Donnelly, 2000) and a multivariate analysis method called DAPC (Jombart & Balloux, 2009). These two different analyses were done because STRUCTURE uses allele frequency and LD information from the dataset directly assuming the HWE equilibrium, while DAPC does not consider a particular population genetics model outlining the genetic differentiation between and within groups (Jombart & Balloux, 2009).

Individual-based clustering analyses were performed with two datasets: one including East Timor, Indonesia, and Mozambique, and the second including just East Timor and Indonesia. The Bayesian model-based clustering algorithm in STRUCTURE v.2.3.4 was used to identify genetic clusters assuming admixture and correlated allele frequencies without using population information. In the first approach, including East Timor, Indonesia, and Mozambique, analyses were set for a burn-in period length to 100,000 followed by 1,000,000 MCMC iterations with *K*-values set from 1 to 14 with 10 runs computed for each *K*. For the second approach, including just the cashew populations in East Timor and Indonesia, the same settings were followed by configuring *K*-values from 1 to 12 with 10 runs in each *K*. Structure Harvester v0.6.94 (Earl & Bridgett, 2012) was then used to calculate Δ*K ad hoc* statistics from Evanno, Regnaut & Goudet (2005), which was then used to estimate the most likely *K*-value. CLUMPP v1.1.2 (Jakobsson & Rosenberg, 2007) was used to calculate the average replicate runs for the selected *K*-value to prevent problems with multimodality and label switching between iterations of STRUCTURE runs. CLUMPP results were then plotted using DISTRUCT v1.1 (Rosenberg, 2004).

The DAPC was performed in R (R Core Team, 2021) using the *adegenet* v1.3.1 package (Jombart, 2008) and the relative frequency of the alleles as the dataset, since presence/absence data may overlook relevant patterns in allele frequency. The *find.clusters* function was used to find the ideal *K*-value based on Bayesian information criterion (BIC) scores, maintaining default parameters and retaining all principal components (PCs). Cross validation was done using the *xvalDapc* function to determine the optimal number of PCs to retain in the Discriminant Analysis (DA). The generated DAPC script is available at Figshare (Barros et al., 2022).

## Results

### SSR genotyping and statistics

All 16 SSRs were tested in singleplex reactions at the estimated optimal annealing temperature, and grouped into 4-plex reactions based on the results of this initial quality assessment (Table 2). The allele profiles were clear and easy to score for all 16 SSR loci. No errors were detected in the genotypic data matrix, indicating there were no potential errors associated with stuttering bands or large allele dropout in the SSRs screened. In 60 of the 224 locus comparisons, the frequencies of the null alleles were higher than 0.20 in markers mAoR33, mAoR12, mAoR29, and mAoR41, so subsequent analyses were completed with the remaining 12 SSRs (Table 3). Deviations from the Hardy-Weinberg Equilibrium (HWE) were observed in most loci except for mAoR48, mAoR42, mAoR3, mAoR17, and mAoR35, with 84 statistically significant locus-population combinations (*p* ≥ 0.05); after a sequential Bonferroni correction, only two loci (mAoR3 and mAoR35) displayed significant deviations, matching 33 of the 168 locus-population combinations (Table S1). All 12 loci were in linkage equilibrium after the Bonferroni correction, indicating they were non-correlated, and that the alleles were independently segregated and inherited (Table S2). Negative fixation index (*F*) estimates were observed in two loci, mAoR17 (−0.03) and mAoR7 (−0.04) (Table 3), which indicates more heterozygotes than expected or other population structure complexities.

**Table 3:** Marker’s diversity measurements. The level of genetic diversity of each SSR marker was described with the parameters using:of total number of alleles, polymorphism information content (PIC), gene diversity (expected heterozygosity, H_e_), observed heterozygosity (H_o_), and the inbreeding/fixation coefficient (*F*). About 207 individuals from 14 populations were analyzed for each locus.

**Locus**	**Allele number**	**PIC**	**H**_**e**_	**H**_**o**_	***F***
mAoR48	11	0.64	0.69	0.49	0.2
mAoR6	17	0.68	0.71	0.55	0.11
mAoR17	19	0.83	0.84	0.73	−0.03
mAoR7	15	0.77	0.8	0.64	−0.04
mAoR11	16	0.66	0.69	0.47	0.19
mAoR3	25	0.74	0.76	0.48	0.24
mAoR42	14	0.65	0.69	0.59	0.03
mAoR52	14	0.67	0.7	0.57	0.05
mAoR2	4	0.48	0.57	0.44	0.13
mAoR35	9	0.62	0.65	0.28	0.48
mAoR47	7	0.63	0.69	0.49	0.06
mAoR16	6	0.4	0.45	0.34	0.03
Total	157				
Mean	13.08	0.65	0.69	0.51	0.12

### Genetic diversity estimates

Overall, a total of 157 alleles were detected in the 207 individual cashew trees analyzed (Table 3). All SSR loci screened were polymorphic, indicating more than one allele in all populations. The total number of alleles per locus ranged from 4 (mAoR2) to 25 (mAoR3) with an average of 13.08 alleles per locus (Table 3). Polymorphic Information Content (PIC) values ranged from 0.40 (mAoR16) to 0.83 (mAoR17) with a mean value of 0.67 (Table 3). In the 12-loci dataset, the observed heterozygosity (H_o_) varied from 0.25 (mAoR35) to 0.73 (mAoR17) with a mean of 0.40 (Table 3), and the expected heterozygosity (H_e_) varied between 0.45 (mAoR16) and 0.84 (mAoR17).

In a population genetic diversity analysis (Table 4), East Timor had an average of 10.67 alleles and Indonesia had the lowest average number of alleles with 3.42. Expected heterozygosity (H_e_) was 0.67 in East Timor, 0.56 in Indonesia, and 0.71 in Mozambique, while H_o_ was 0.51, 0.47, and 0.54, respectively. All populations presented positive F values, ranging from 0.12−0.25. The populations with the highest number of alleles were observed in East Timor, specifically in the ETK population which had an average of 5.17 alleles, followed by ETR3 with 5.08 alleles, while ETBAT and ETV had an average of 2.42 and 2.25 alleles, respectively. Comparing the SSR allele numbers of the populations screened identified which population harbored more allelic diversity. When analyzing this parameter, ETK and ETTR3 were the populations identified with the most allelic diversity (Na, Table 4). The highest expected heterozygosity (H_e_) was observed in the ETK population, and the lowest was observed in the ETV population. Thre highest heterozygosity (H_o_) was observed in the ETTR1 population, and the lowest was observed in the ETFA population. The fixation index (*F*) was positive in all populations except for ETSAN and ETV; the positive values in all other populations may indicate genetic stability and a higher rate of inbreeding. The absence or existence of private alleles is important for identifying a specific genetic signature, as private alleles are alleles that are unique to a population. A high number of private alleles (6) were observed in East Timor. Two private alleles were observed in Mozambique, while no private alleles were detected in the Indonesian cashew populations.

**Table 4:** Genetic diversity indices derived from a country analysis and a population approach scheme. Countries: Mozambique (MZ, 2 populations), Indonesia (IND, 1 population), and East Timor (ET, 11 populations); Total number of populations: 14. Sample size (N). Genetic diversity indices for each group were assessed using: mean alleles per locus (Na), expected heterozygosity (H_e_), and observed heterozygosity (H_O_) with corresponding standard deviation (SD) values, private alleles (*P*_A_), and an inbreeding/fixation coefficient (*F*).

	N	Na	Na SD	H_e_	H_e_ SD	H_o_	H_o_ SD	*P*_A_	*F*
**Countries**									
East Timor (ET)	164	10.67	4.96	0.670	0.033	0.506	0.012	6	0.24
Indonesia (IND)	16	3.42	1.93	0.561	0.063	0.465	0.037	0	0.12
Mozambique (MZ)	27	5.50	2.28	0.711	0.028	0.535	0.028	2	0.25
Mean	69	6.53	3.06	0.647	0.0413	0.502	0.0257	–	0.20
**Populations**									
ETK	17	5.17	1.75	0.67	0.04	0.56	0.04	1	0.14
ETNA	14	3.75	1.14	0.65	0.03	0.56	0.04	0	0.10
ETTR1	14	3.83	1.53	0.65	0.04	0.57	0.04	0	0.06
ETTR2	14	3.67	1.72	0.58	0.06	0.49	0.04	0	0.11
ETTR3	16	5.08	2.54	0.59	0.06	0.51	0.04	0	0.07
ETSU	16	3.58	1.44	0.65	0.04	0.46	0.04	0	0.26
ETSAN	18	3.58	1.83	0.57	0.05	0.57	0.03	0	−0.04
ETMA	14	3.00	0.85	0.57	0.03	0.46	0.04	0	0.14
ETBAT	13	2.42	0.79	0.48	0.05	0.45	0.04	0	0.01
ETFA	14	2.75	0.75	0.51	0.05	0.43	0.04	0	0.10
ETV	14	2.25	0.62	0.45	0.06	0.45	0.04	0	−0.09
IND	16	3.42	1.93	0.56	0.06	0.47	0.04	0	0.12
MZB	12	4.33	1.50	0.69	0.03	0.51	0.04	1	0.23
MZD	15	3.50	1.17	0.65	0.03	0.56	0.04	0	0.12
Mean	14.79	3.60	1.40	0.59	0.05	0.50	0.04	–	0.09

### Population structuring analyses

#### Using genetic distance to estimate relationships between populations

**Figure 2:**
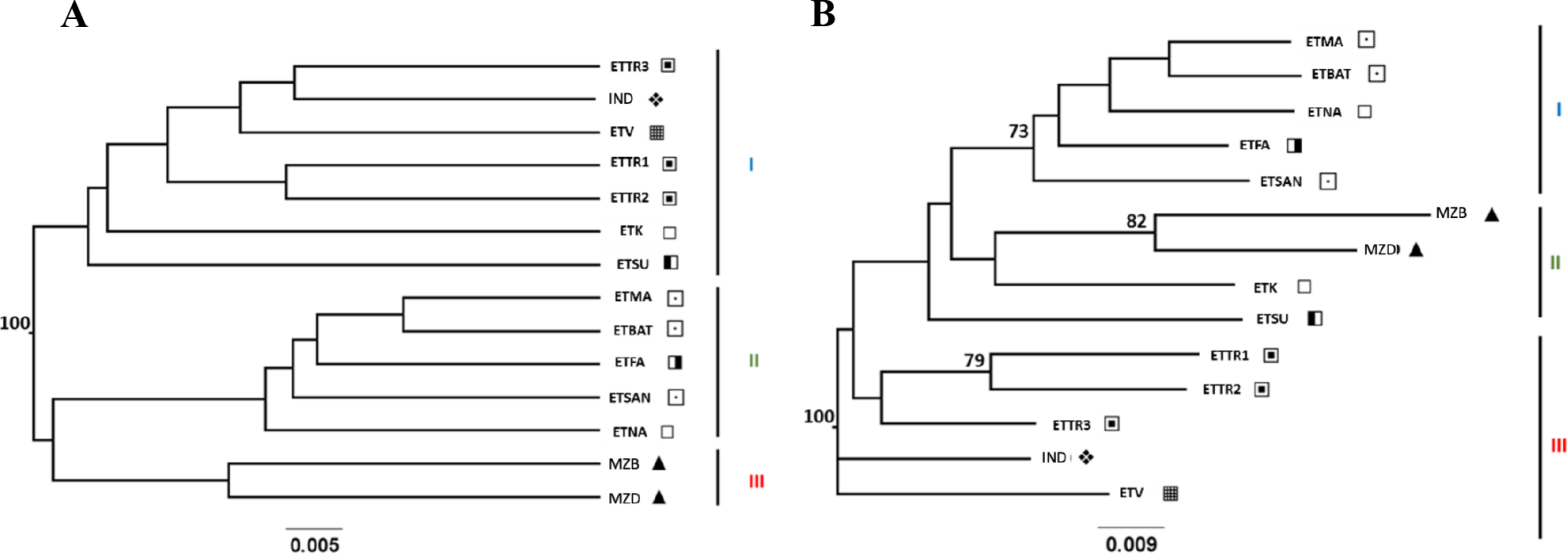
UPGMA (A) and NJ (B) trees generated from *FreeNA* using matrix *DC*^INA^ of the East Timor, Indonesia, and Mozambique dataset. 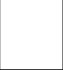
 Manatuto, 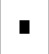 Bobonaro, 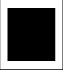 Baucau, 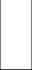 Cova Lima, 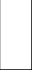 Manufahi, 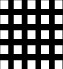 Viqueque, 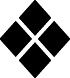 Indonesia, ▴ Mozambique.

**Figure 3:**
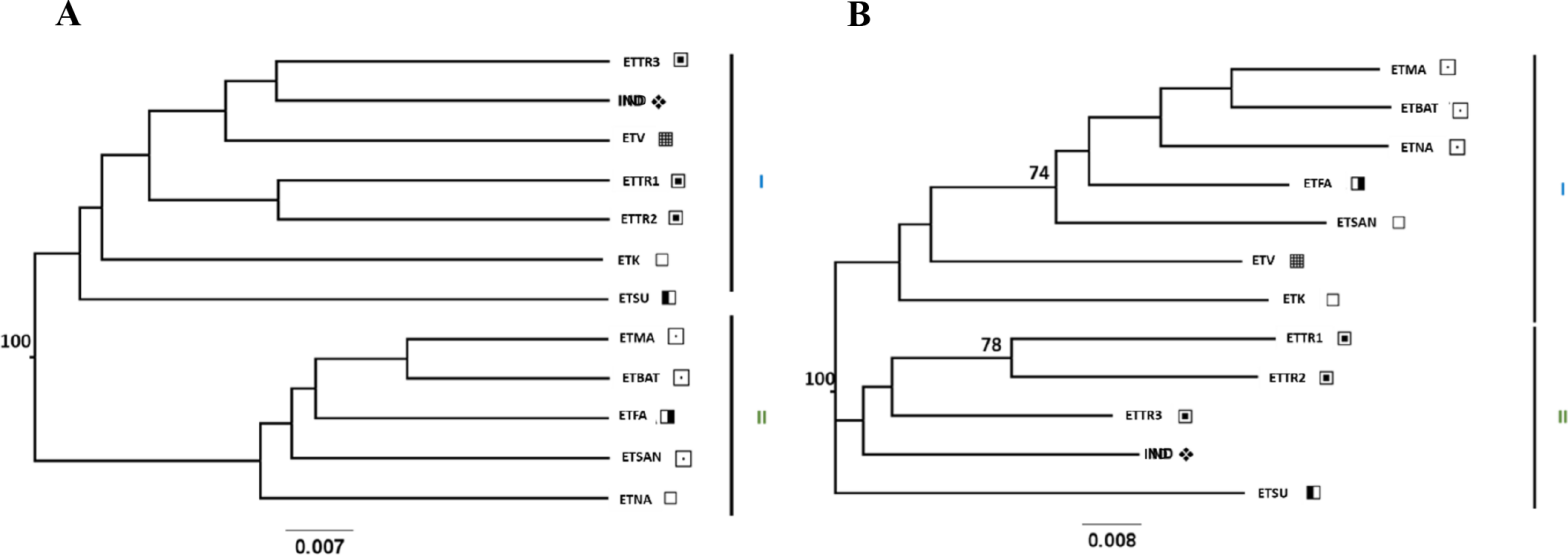
UPGMA (A) and NJ (B) trees generated from *FreeNA* using matrix *DC*^INA^ presenting the East Timor and Indonesia populations. 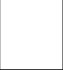
 Manatuto, 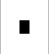 Bobonaro, 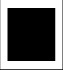 Baucau, 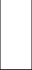 Cova Lima, 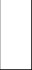 Manufahi, 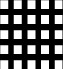 Viqueque, 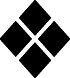 Indonesia.

UPGMA and NJ trees were built using Nei’s *D* and *DC*^INA^ (*FreeNA*) genetic distances across accessions screened for East Timor, Indonesia, and Mozambique (Table S3), and an analysis narrowed to East Timor and Indonesia (Table S4) was also performed. Similar tree topology structures were observed with both Nei’s *D* (Figs. S1 and S2) and *DC*^INA^ (Figs. 2 and 3) matrices, indicating a reliable topology regardless of the algorithms used for genetic distance. Because of the similarity between the two algorithmic results, only the trees derived from *DC*^INA^ distance matrices are presented in Fig. 2 (East Timor, Indonesia, and Mozambique analyses) and Fig. 3 (East Timor and Indonesia). In the UPGMA- (Fig. 2A) and NJ- (Fig. 2B) derived trees, three clusters were identified: one cluster (I) that included the ETTR populations (ETTR1-3) from Baucau, Viqueque (ETV), Cova Lima (ETSU), and the ETK population from Manatuto, grouped with Indonesia (IND); a second cluster (II) that included the remaining East Timor populations from Manatuto (ETNA), all populations from the Bobonaro district (ETMA, ETBAT, ETSAN), and the Manufahi population (ETFA); and the third (III) cluster including just the populations from Mozambique (MZB and MZD). These results support two different genetic clusters within East Timor: one including only East Timor populations (Cluster III) and the other with a high genetic similarity with the Indonesian cashew population (Cluster II). In the NJ tree (Fig. 2B), the dendrogram derived from the *DC*^INA^ distance matrix also presented three different clusters: cluster one included the cashew populations from Mozambique, as observed in the UPGMA (Fig. 2A); the second cluster included the Bacau, Viqueque (ETV), Cova Lima (ETSU), and Indonesian populations; and the third cluster included the Bobonaro populations (ETMA, ETBAT and ETSAN) and the remaining East Timor populations (ETFA, ETNA and ETK).

When observing the cashew populations without including those from Mozambique (Fig. 3), the two clusters were similar to clusters I and II observed in the UPGMA, as shown in Fig. 2.

Conversely, the two genetic distance algorithms (Nei’s *D* distance, Fig. 3B, Figs. S2A and S2B) produced dissimilar NJ-generated trees to those derived from both the three-country dataset (Fig. 2A, Fig. S1A) and the East Timor/Indonesia dataset (Fig. 3A, Fig. S2B) from the UPGMA, indicating complex population structuring.

#### Analysis of molecular variance

AMOVA results from all three countries (MZ, IND, ET) showed that molecular variation was mainly found within individual cashew trees (76%), whereas variation among populations and among individual trees explained 13% of the total genetic differentiation, in both cases (Table 5). AMOVA results including only East Timor and Indonesian cashew populations were similar, with genetic differentiation within individual trees (73%) also higher than among individual trees (14%) or among populations (13%).

**Table 5:** AMOVA results including fixation indices *F*_CT_, *F*_SC_, and *F*_ST_.

**Source of variation**	**df**	**Sum of Squares**	**Variance components**	**Variation (%)**	**Fixation indices**
**Countries (MZ, IND, ET)**					
Among pops	13	168.537	*V*_a_ = 0.34692	12.6	*F*_CT_ = 0.128^*^
Among individuals	193	523.90	*V*_b_ = 0.30894	11.22	*F*_SC_ = 0.126^*^
Within individuals	207	434.00	*V*_c_ = 2.09662	76.17	*F*_ST_ = 0.238^*^
**Countries (ET, IND)**					
Among pops	11	91.172	*V*_a_ = 0.16546	12.97	*F*_CT_ = 0.273^*^
Among individuals	168	288.058	*V*_b_ = 0.12973	14.4	*F*_SC_ = 0.165^*^
Within individuals	180	221.00	*V*_c_ = 1.22778	72.63	*F*_ST_ = 0.129^*^

**Notes.**

*V*_a_ variance among populations *V*_b_ variance within populations *V*_c_ variance within individuals

The genetic differentiation between countries and East -Timor vs Indonesia populations is denoted as *F*_CT_, among individuals within populations as *F*_SC_ and within individuals as *F*_ST_.

* *p* < 0.001

#### Individual-based clustering using Bayesian and a multivariate discriminant analysis to uncover population structure

Two different approaches were used to identify individual clusters in East Timor, using Indonesian cashew populations as the outgroup: (1) covering all populations from East Timor, Indonesia, and Mozambique, and (2) excluding Mozambique.

**Figure 4:**
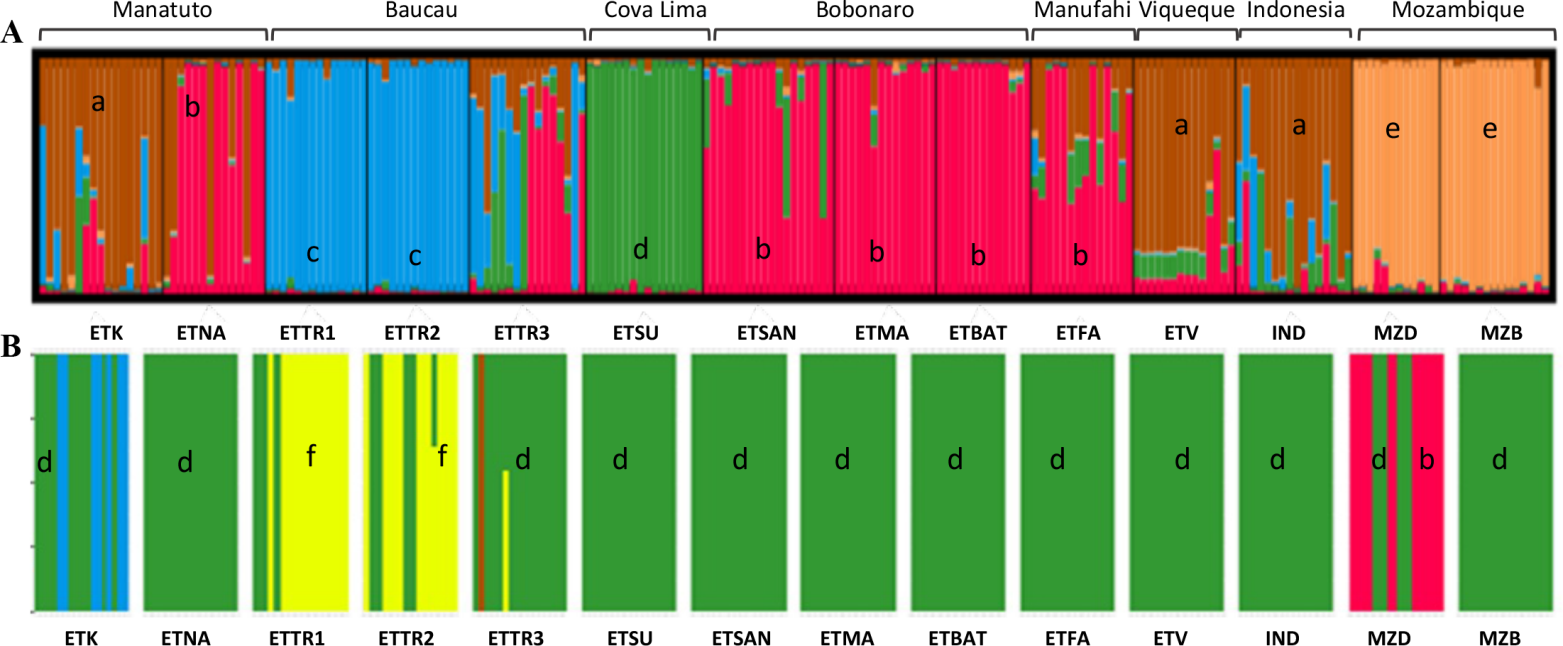
Clustering based on SSR data of the optimal *K*-means using STRUCTURE (A) for populations in Mozambique, East Timor, and Indonesia; and DAPC analyses of *K* = 5 (B). a, brown; b, red; c, blue; d, green; e, orange; f, yellow.

In the first approach, STRUCTURE was run considering the highest range of clusters conceivable (*K* = 1–15). This analysis assigned *K* = 5 as the optimal number of groups based on the Δ*K* method (Fig. S3) outlined by Evanno, Regnaut & Goudet (2005). According to the results when including populations from East Timor, Indonesia, and Mozambique, in *K* = 5, Mozambique was grouped in a single cluster (orange cluster; Fig. 4A); Manatuto populations were grouped into two principal clusters (brown and red); Baucau populations were mostly in one cluster (blue), except for ETTR3 that included a mix of all clusters except the cluster from Mozambique; Cova Lima was grouped in a single cluster (green cluster); Bobonaro populations were grouped into the red cluster; and the Manufahi populations were mostly grouped into the red cluster sharing genetic flow with populations from Viqueque and Indonesia, which were grouped into two clusters (brown) (Fig. 4A).

The DAPC analysis was done without assigning an *a priori* group. For the first dataset, the clustering analysis determined that *K* = 10 was the one with the best combination of mean and 95% CI of BIC (Fig. S4). However, there was no “elbow” effect observed, but a large plateau in the number of clusters with a significant overlap among confidence intervals for the nearby number of clusters. *K* = 5 was identified as the optimal number of clusters for later analysis to produce results comparable to the STRUCTURE results (Fig. 4B). Using the *xvalDAPC* function, 40 PCs (highest successful assignment: 88.2%, with the lowest mean squared error: 0.178) were retained and four discriminant functions, conserving 87.5% of the variance (Fig. S5).

Cross validation was performed using the *xvalDapc* function with the outcome being the number of PCA axes retained against the proportion of successful outcome predictions. The results showed 40 PCA axes were retained (considering the highest successful assignment: 88%, with the lowest mean squared error: 17%) and two discriminant functions (explaining 87.5% of cumulative variance) for inferring the five genetic clusters (Fig. S6). The loading plots from both discriminant functions identify which variables (*i.e.,* alleles/loci) contributed the most for the five genetic clusters. Both DAPC results (*K* = 9 and *K* = 10) identified the same variables responsible for cluster assemblage (172 (mAoR6), 328 (mAoR17), 174 (mAoR35), 170 (mAoR47)), which highlights the importance of these alleles for cluster discrimination (Figs. S6 and S9).

**Figure 5:**
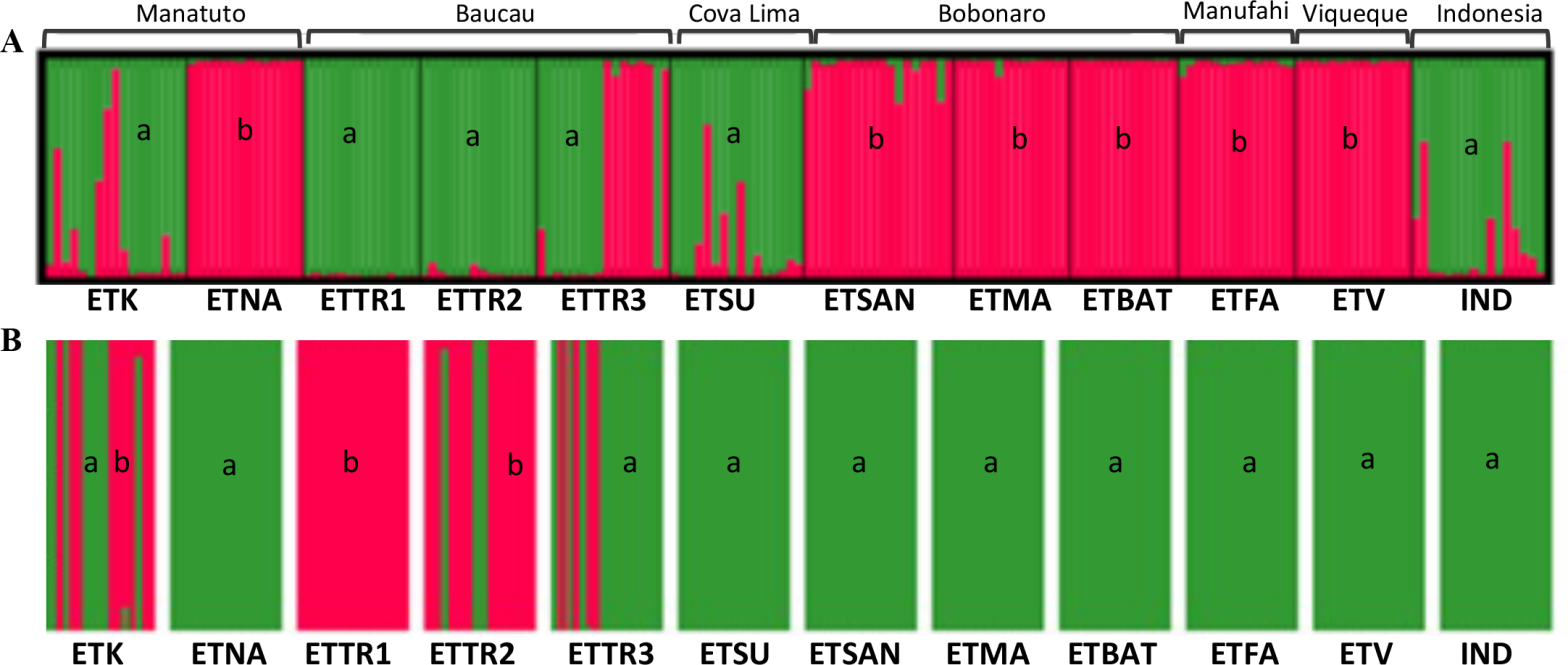
Optimal *K*-means individual-based clustering using STRUCTURE (*K* = 2, (A) for populations in East Timor and Indonesia and DAPC analyses of *K* = 2 (B). a, green; b, red.

For the Bayesian analysis of the second approach, which included populations from East Timor and Indonesia, STRUCTURE was run considering the highest range of clusters conceivable (*K* = 1 − 13). This analysis identified *K* = 2 as the optimal number of groups (Fig. S8) based on the methods outlined by Evanno, Regnaut & Goudet (2005), with no alternative ideal *K*. Considering the high Δ*K*-values displayed for *K* = 2 (Fig. 5), two clusters were observed: the first cluster, in green, includes cashew populations in Baucau, Cova Lima, Manatuto, and Indonesia; and the second cluster, in red, includes cashew populations in Bobonaro, Manufahi, and Viqueque (Fig. 5A). For the DAPC analysis with *K* = 2 (Fig. 5B), the cross-validation retained 20 PCA axes (considering the highest successful assignment: 93.5%, with the lowest mean squared error: 10%) and two discriminant functions (explaining 96.4% of the cumulative variance) for inferring the two genetic clusters. For *K* = 2, there were differences between the STRUCTURE and DAPC analyses. The DAPC results showed a genetic diversity in populations from Baucau that was not shared with Indonesian populations, while the Bobonaro, Manufahi, and Viqueque populations showed a common allelic diversity with Indonesia (Fig. 5B), contrary to the STRUCTURE optimal clustering results (Fig. 5A). These analytical inconsistencies may be related to different pre-requisites associated with both methods: STRUCTURE assumes that markers are not linked and that populations are panmictic (Pritchard, Stephens & Donnelly, 2000), while DAPC is better for populations that are clonal or partially clonal. In this case, STRUCTURE provides a more realistic observation of the genetic diversity of cashew populations, given the panmictic nature and absence of clonal populations in cashew varieties.

## Discussion

A comprehensive sampling of cashew orchards in East Timor was conducted by assessing and characterizing the genetic diversity and population structuring of 11 cashew populations from major cashew-producing districts in the country. A total of 207 individual cashew trees belonging to 14 cashew populations from three tropical countries (East Timor, Mozambique, and Indonesia), using Indonesia and Mozambique populations (*n* = 3) as outgroups, were screened using 16 cashew-specific microsatellites.

### Cashew diversity assessment

Fourteen different cashew populations were first genotyped with 16 SSRs, and a quality assessment of the SSRs was also performed (Table 3). Four loci (mAoR12, mAoR33, mAoR41, mAoR29) were discarded due an excess of null alleles in almost all populations (null allele frequency >0.20), despite being polymorphic across the cashew populations analyzed identifying them as informative markers for future diversity analyses. Subsequent genetic diversity indices and population structuring analyses were performed using 12 loci. The obtained PIC values were high (average PIC = 0.65), indicating high informativeness. The PIC values obtained in this study were also higher than the average PIC value of 0.33 found in a recent study using 21 cashew SSRs (cSSR, Savadi et al., 2021). These differences in PIC values might be because of the distinct plant material sources used in this study compared to former reports, which may influence the number of alleles detected at each SSR locus, though the lower number of SSR loci used in this study may also impact PIC values. When analyzing the presence of null alleles and their effect on population structure, only 60 of the 224 locus-comparisons harbored null alleles with a frequency higher than 0.20. The preceding analysis showed that the SSR loci used in this study were suitable for a downstream genetic diversity analysis.

The genetic diversity observed in this study was high compared to other cashew studies. More alleles were found within East Timor, which can be partially explained by a higher sampling effort but also may be indicative of a reservoir of alleles associated with traits conferring tolerance to various biotic and abiotic stresses or to local adaptation conditions. The study results showed a higher allelic richness in East Timor cashew populations (Na = 10.67, Table 4) than in populations in Mozambique (Na = 3.42) and Indonesia (Na = 3.42), which highlights the high genetic diversity in East Timor. Future studies including more populations from Mozambique and Indonesia could corroborate if cashew populations in East Timor have higher allelic richness. Within East Timor, the ETK population from the Manatuto district had the highest allelic richness (Na = 5.17), followed by ETTR3 from the Baucau district, with ETV from Viqueque having the lowest allelic diversity (Na = 2.25, Table 4). Less observed diversity in Viqueque is likely because cashew orchards are less frequent in the Viqueque district compared with the Baucau and Manatuto districts, where several orchards have been planted over the last 20 years. The populations analyzed in this study might reflect long-term genetic diversity that can be exploited in a breeding program to improve yield and nut quality (Kouakou et al., 2020). Moreover, identifying trees with high allelic richness is necessary for conserving the cashew germplasm (Bataillon, David & Schoen, 1996). The number of private alleles found within accessions is an important measurement of diversity since these alleles represent genotypic-specific allelic build-up. Contrasting with previous SSR studies in cashew populations (Savadi et al., 2021), private alleles were detected in our study, which may allow for the configuration of a unique genetic signature of East Timor cashew populations, using different allele frequencies or the unique alleles in each population. This is of major importance in an agricultural crop like cashew, where nuts are frequently exported and processed outside the producing countries, as it may lead to the valorization of a domestic product with a geographical origin.

The genetic diversity and population structuring of the cashew population in East Timor was the primary focus of this study. From 2006 to 2020, only a few papers on genetic cashew diversity or population structuring were published, especially in Southeast Asia and the West Africa region. In comparison with a study done in Benin (West Africa) where a lower genetic diversity (Shannon index = 0.04) was observed in 60 cashew morphotypes using eight SSR markers (Sika et al., 2013), a much higher genetic diversity was observed in the East Timor populations. In Brazil, wild Brazilian populations of cashew were studied (Dos Santos et al., 2019), and the genetic diversity in wild populations was higher than in domesticated ones, despite a weak distinction between wild and domesticated groups and with no correlation between genetic and geographical interpopulation distance. In Cô te d’Ivoire, cashew genetic diversity was studied using SSRs and the resultsnrevealed an overall heterozygosity deficit and a high intra-population genetic diversity among the screened cashew populations (Kouakou et al., 2020), which is in accordance with the AMOVA results of this study where the most genetic diversity was depicted within populations. The results obtained from this study also agree with a recent study from Burkina Faso, where a substantial amount of genetic diversity was observed across the 18 cashew accessions screened with four SSRs (Moumouni et al., 2022).

#### Population structuring in the East Timor germplasm

The AMOVA showed that most of the genetic diversity lies within populations with little diversity present between populations within East Timor or between populations in each country. The most diversity was observed within accessions for both groupings (all three countries and only East Timor and Indonesia) suggesting a high gene flow between populations, which is in accordance with previous studies (Freitas & Paxton, 1996). Cashew is an allogamous species favoring cross-fertilization (Freitas & Paxton, 1996), allowing intraspecific hybridizations and heightening genetic variation. Overall, outcrossing species tend to have higher genetic variation within populations, whereas selfing species, or species with a mixed mating system, have less genetic variability (Nybom, 2004). Since cashew is an outcrossing species, negative to low inbreeding coefficients (*F*) were observed, which agrees with former studies (Freitas & Paxton, 1996; Layek et al., 2021).

A population structuring analysis revealed that the distribution of genetic diversity did not follow a clear geographic trend, despite a well-defined clustering observed between cashew populations in Mozambique with the remaining populations in East Timore and Indonesia. The unique clustering attributed to the cashew populations in Mozambique may be because the cashew populations chosen in Mozambique may be different varieties than the ones used by farmers in both Indonesia and East Timor. There may also be differences because of the geographical isolation of the island countries, Indonesia and East Timor, compared to continental Mozambique. When narrowing the analysis to East Timor, a complex population structuring was observed, linked to district-associated genetic diversity. Within the country, the Viqueque, Manufahi, and Bobonaro districts displayed unique allelic diversity, while Baucau, Cova Lima, and Manatuto had a high genetic similarity with the Indonesian cashew population. These dissimilar results when including *vs* excluding the cashew populations in Mozambique highlights the complex genetic diversity of cashew trees in the context of a continental (Mozambique) country where orchards are older and have incorporated improved cashew varieties. These results are in accordance with previous results in India (Archak et al., 2009), where cashew genetic diversity lies within geographical populations and where the sharing of allele frequencies among populations does not translate into an in-country population structuring.

The clustering of cashew populations in this study had an uncommon, yet existing relationship with geographical region under a district-wise distribution, which is contrary to previous reports in India (Archak et al., 2009), where no relationship with the geographic region was observed. One of the reasons for a district-specific genetic diversity pattern may be related to seed availability and farmer preference by district, with varieties selected based on local performance and yield. Considering that few cashew varieties have been introduced in East Timor, this genetic diversity distribution may be associated with an inter-exchange of seed material adapted to similar ecological conditions. Despite the high genetic diversity observed and attributed to the high heterozygosity, allogamous nature, and high gene flow found in cashew (Mitchell & Mori, 1987; Borges, 2018), in certain East Timor districts, such as Bobonaro, Viqueque, and Manufahi, the dissimilar genetic clustering from the remaining districts suggests a diverse genetic build-up, which could be attributed to different cashew varieties being planted.

The fixation index *F* (also called the inbreeding coefficient) exhibits values from −1 to +1. Values close to zero are expected under random mating, while substantial positive values indicate inbreeding or undetected null alleles (Monteiro et al., 2016). Negative values denote an excess of heterozygosity due to negative assortative mating or heterozygote selection. Overall, positive *F*-values were observed across all populations (Table 4), revealing that populations are at or near the Hardy-Weinberg equilibrium, further supported by the lower observed heterozygosity values under the HWE compared to the expected values (Table 4). Among the cashew populations collected (associated with different geographic regions), the inbreeding coefficient was lowest in the northern Bobonaro district (ETSAN, *F* =  − 0.04; ETMA, *F* = 0.14; ETBAT, *F* = 0.01, Table 4) and in the southern Viqueque district (ETV, *F* =  − 0.09), possibly because growers from both regions exchange seeds with the other regions. In contrast, the Cova Lima district (south) showed the highest inbreeding coefficient (*F* = 0.26). Both the Bobonaro and Viqueque districts displayed a significant share of planting material exchange with Bobonaro getting seeds from Indonesia and Viqueque getting seeds from Australia. In the studied populations, positive *F* values were observed, which may indicate some level of inbreeding. The difference between expected heterozygosity and observed heterozygosity might be due to evolutionary factors (such as inbreeding) or internal genetic factors (such as gene incompatibility). The introduction of new cashew seeds for new orchards might be another explanation; when establishing new cashew orchards, some producers used seeds from a single tree with good traits (high yield and large nuts). Moreover, according to Waples (2015), the genetic makeup of a given population can vary over time in response to evolutionary forces that affect the heterozygosity of the population relative to the Hardy-Weinberg equilibrium.

Considering the 11 populations studied from East Timor, population differentiation (*F*_ST_ = 0.129, Table 5) was relatively moderate in comparison to a previous study of cashew trees in Côte d’Ivoire (Kouakou et al., 2020) where a low differentiation was indicative of a common origin. However, moderate differentiation could also be associated with the lower number of molecular markers used in this study (12 SSRs *vs* 18 SSRs in Kouakou et al., 2020). In India, a similarly low genetic diversity among cashew trees was associated with cashew trees being a relatively recent introduction to the country (Archak et al., 2009), which is also reported in other countries (*e.g.*, Benin, Kouami et al., 2020). In East Timor, there are two possible explanations for the cashew genetic diversity observed in this study: (i) multiple cashew varieties were introduced from the Bobonaro and Viqueque districts, these regions being regarded as the cashew introduction hotspots in East Timor; or (ii) new cashew varieties were introduced in the other districts in East Timor.

The moderate genetic variability observed among the populations screened could also be due to the relatively recent introduction of cashew trees into East Timor on an evolutionary timescale and the allogamous nature of cashew, resulting in high gene flow and exchange of genetic material. The unexplored, yet genetically diverse accessions from East Timor could be used in future cashew breeding programs to improve yield, quality, and other traits.

#### East Timor as an unexplored, yet important source of genetic cashew diversity

The wide distribution of cashew in its primary center of diversity, Brazil, has been attributed to mature fruit floating down water currents as well as bats dispersing cashew seeds (Johnson, 1973). However, outside Brazil, cashew distribution has been attributed primarily to anthropogenic efforts, rather than through natural means (Archak et al., 2009). The extent and distribution of genetic diversity, as revealed by the present study, provide some clues to cashew introduction and expansion in East Timor. The existence of substantial overall genetic diversity and two distinct genetic population groupings in East Timor point to multiple introductions comprising different founder populations. These results are in accordance with a recent report in Burkina Faso that suggests several historical cashew introductions with well-performing cashew cultivars disseminated through the same route across producing areas (Moumouni et al., 2022). If the introduction occurred as a one-time event, the founder effect would have been reflected with a low genetic diversity. Cashew expansion in East Timor has been promoted mainly through the introduction of different varieties from Brazil, Indonesia, Australia, as well as a so-called “native” cashew. According to information obtained from the National Directorate of Industrial Crops and Agribusiness (Ministry of Agriculture, Forestry and Fisheries (MAFF) of East Timor), the Portuguese introduced the cashew to East Timor (ET) in the 18th century, where it was planted ornamentally in various districts. Since the establishment of cashew as an industrial crop in the 1990s (Buss & Ferreira, 2010; Do Céu Guterres, 2017), there has been no standard implemented for which varieties to include in orchards. In the Cova Lima district, cashew orchards were implemented in the 1990s and few new cashew varieties have been introduced. In the Manatuto and Baucau districts, cashew orchards were planted with accessions known for higher yield and that were better-adapted to local agro-ecological conditions. During fieldwork in the Bobonaro and Viqueque districts, land farmers reported that several cashew accessions were being introduced, namely from Indonesia and Australia; the different genetic clusters in these districts may be associated with these introductions based on the preferences of the farmers, thus shaping the current East Timor cashew genetic diversity panorama.

Considering India’s importance as the place cashew was first introduced as a commercial crop in Asia (Singh, 2018), future studies on genetic cashew diversity in East Timor should include Indian cashew populations to help explain the historical introduction of cashew trees in the country. In East Timor, a relatively significant genetic diversity for an introduced species was observed, supporting the possibility of cashew being introduced repeatedly over time. Similar observations have been reported in India (Archak et al., 2009). Contrary to the results of this study, a significant level of redundancy (homogenous group) was reported within the Nigerian cashew germplasm (Aliyu, 2012), highlighting a narrow genetic diversity. Results gathered from East Timor are of major importance, since the country aims to invest in cashew nut exports. Assessing the genetic diversity of cashew trees in East Timor will help define a valorized genetic signature of cashew nuts from East Timor. The results of this study are, thus, also relevant to the local government of East Timor, since the country would compete directly with major world cashew nut exporters, such as India and Vietnam.

This study provides useful information on the genetic diversity of cashew populations in a largely understudied country, where cashew nuts are becoming an important export. The genetic diversity results from this study identify a genetic cashew diversity hotspot. Cashew has been implemented under a monoculture system and cashew orchards have been increasing to meet global market needs (Monteiro et al., 2017). With the rising potential of pests and diseases (Monteiro et al., 2015; Monteiro et al., 2017), the current narrow genetic diversity of cashew orchards is a major concern to the future sustainable production of cashews. To ensure a solid future, cashew varieties with improved agronomic traits, such as higher yield as well as biotic and abiotic stress tolerance should be introduced. Also, the increase of the genetic diversity of on-farm orchards at the short run and the identification of genetic resources for the development of cashew genetic management should not be neglected.

## Conclusions

The results of this study showed a higher allelic richness in cashew populations in East Timor than in populations in Mozambique and Indonesia, reinforced by the presence of private alleles. Genetic diversity was observed within populations, in accordance with former studies. An analysis of population structuring revealed that the genetic diversity seemed to follow a geographic trend, with a well-defined cluster observed in cashew populations in Mozambique with another observed in the cashew populations in East Timor and Indonesia. Within East Timor, two district-associated genetic diversity clusters were observed, which may point to multiple points of historic cashew introduction. This study provides useful information on genetic cashew diversity hotspots, which can be used to improve genetics and characterize new cashew types in future breeding efforts. In East Timor, cashew nuts are becoming an important tradeable agriculture product, and the genetic diversity observed in this study identified cashew agrobiodiversity hotspots. The findings of this study are also applicable to the development or preservation of genetic cashew resources in East Timor. Considering that East Timor is looking to compete in the global cashew market, these study results are also relevant to the creation of an in-country genetic signature to increase the market value of cashew nuts from East Timor.

##  Supplemental Information

10.7717/peerj.14894/supp-1 Table S1 Hardy-Weinberg equilibrium (HWE) test for each locus-population combination using GenePop v4.5 Statistical significance was assessed by running 10,000 iterations Monte Carlo Markov Chain (MCMC) test. *p*-values were corrected by multiple comparisons applying a sequential Bonferroni correction (*p* < 0.000298, [0.05/168]), *p* < 0.05, light blue; *p* < 0.000298, dark blue.)

10.7717/peerj.14894/supp-2 Table S2 Linkage Disequilibrium (LD) test for each locus-population combination using GenePop v4.5 Statistical significance was assessed by running 10,000 iterations Monte Carlo Markov Chain (MCMC) test. *p*-values were corrected by multiple comparisons applying a sequential Bonferroni correction (*p* < 0.000298, [0.05/168]), *p* < 0.05, light blue; *p* < 0.000298, dark blue if present).

10.7717/peerj.14894/supp-3Table S3Pairwise *F*_*ST*_ (lower-left matrix) and ${F}_{ST}^{ENA}$ (upper-right matrix) between all populations in East Timor, Indonesia, and MozambiqueClick here for additional data file.

10.7717/peerj.14894/supp-4Table S4Pairwise *F*_*ST*_ (lower-left matrix) and ${F}_{ST}^{ENA}$ (upper-right matrix) between all populations in East Timor and IndonesiaClick here for additional data file.

10.7717/peerj.14894/supp-5 Figure S1 UPGMA (A) and NJ (B) trees generated using matrix Nei’s *D* distance representing the cashew populations of East Timor, Indonesia, and Mozambique Manatuto, Bobonaro, Baucau, Covalima, Manufahi, Viqueque, Indonesia and Mozambique.

10.7717/peerj.14894/supp-6 Figure S2 UPGMA (A) and NJ (B) trees generated using matrix Nei’s *D* distance representing the cashew populations of East Timor and Indonesia Legend: Manatuto, Bobonaro, Baucau, Covalima, Manufahi, Viqueque, and Indonesia)

10.7717/peerj.14894/supp-7 Figure S3 STRUCTURE ad hoc statistics retrieved by StructureHarvester using 1 to 15 possible clusters (*K*) STRUCTURE ad hoc statistics retrieved by Structure Harvester using 1 to 15 possible clusters (*K*). Variation of Δ*K* values according to the method outlined by Evanno, Regnaut & Goudet (2005) for populations from East Timor, Indonesia, and Mozambique.

10.7717/peerj.14894/supp-8 Figure S4 DAPC results inference of the number of clusters using the *find.clusters* function with a *K* = 10 (left) and *K* = 5 (right) A *K* value of 10 (the lowest BIC value) represents the best summary of the data for the populations of East Timor, Indonesia, and Mozambique.

10.7717/peerj.14894/supp-9 Figure S5 Scatterplot of the DAPC analysis of *K* = 5 Scatterplot shows the two principal components of the DAPC, and clusters are numbered and displayed by different colors, while dots represent individuals. The 2 Discriminant Functions hereby represented explain 93% of cumulative variance of the dataset.

10.7717/peerj.14894/supp-10 Figure S6 Loading plots of the two discriminant functions following the DAPC analysis with a *K* = 5 (**A**) Allele loading plots from DF1 and (**B**) from DF2, after assigning 0.045 as the threshold.

10.7717/peerj.14894/supp-11 Figure S7 STRUCTURE ad hoc statistics retrieved by Structure Harvester using 1 to 12 possible clusters (*K*) Variation of Δ*K* values according to the methods outlined by Evanno, Regnaut & Goudet (2005) for populations from East Timor and Indonesia.

10.7717/peerj.14894/supp-12 Figure S8 Number of clusters inferred by the DAPC *find.clusters* function with a *K* = 5 and *K* = 2

10.7717/peerj.14894/supp-13 Figure S9 Loading plot of the DF1 following the DAPC analysis with a *K* = 2, after assigning 0.045 as the threshold

## Additional Information and Declarations

### Data Availability

The following information was supplied regarding data availability: The data is available in the Supplementary Files. The genotypic matrix and the DAPC script are available at figshare: Guterres, Lara; Barnabé, João; Monteiro, Filipa (2022): Genotypic matrix for studying the cashew population structure and genetic diversity in East-Timor. figshare. Dataset. https://doi.org/10.6084/m9.figshare.19119041.v3. Barros, Andre; Barnabé, João; Guterres, Lara; Monteiro, Filipa (2022): Script for performing DAPC analysis applied to the study of population structure and genetic diversity in cashew from East-Timor. figshare. Software. https://doi.org/10.6084/m9.figshare.19117889.v3
