# Supplementary material for: East Timor as an important source of cashew (Anacardium occidentale L.) genetic diversity
Source: PeerJ. 2023 Apr 24;11:e14894. doi: 10.7717/peerj.14894 (PMC10135414; doi:10.7717/peerj.14894)
Supplement: Table S1 — Statistical significance was assessed by running 10,000 iterations Monte Carlo Markov Chain (MCMC) test. p-values were corrected by multiple comparisons applying a sequential Bonferroni correction (p < 0.000298, [0.05/168]), p < 0.05, light blue; p < 0.000298, dark blue.) [file peerj-11-14894-s001.docx]

**Supplementary Table S1** Hardy-Weinberg equilibrium (HWE) test for each locus-population combination using GenePop v4.5. Statistical significance was assessed by running 10,000 iterations Monte Carlo Markov Chain (MCMC) test. *p*-values were corrected by multiple comparisons applying a sequential Bonferroni correction (*p*<0.000298, [0.05/168]), *p*<0.05, light blue; *p*<0.000298, dark blue.)

|  | mAoR48 | mAoR6 | mAoR17 | mAoR7 | mAoR11 | mAoR3 | mAoR42 | mAoR52 | mAoR2 | mAoR35 | mAoR47 | mAoR16 |
| --- | --- | --- | --- | --- | --- | --- | --- | --- | --- | --- | --- | --- |
| ETK | 0.0009 | 0.2012 | 0.0000 | 0.1223 | 0.0662 | 0.0000 | 0.0386 | 0.4415 | 0.1147 | 0.0000 | 0.0063 | 0.0000 |
| ETNA | 0.0085 | 1.0000 | 0.0039 | 0.0079 | 1.0000 | 0.0000 | 0.0021 | 0.0005 | 0.3156 | 0.0013 | 0.0004 | 0.0357 |
| ETTR1 | 0.0035 | 0.2665 | 0.0009 | 0.0060 | 0.0575 | 1.0000 | 0.0091 | 0.6355 | 0.2712 | 0.0000 | 0.4874 | 1.0000 |
| ETTR2 | 0.0004 | 0.0017 | 0.0000 | 0.4575 | 0.1235 | 0.0016 | 0.0011 | 0.0000 | 1.0000 | - | 0.2209 | 0.1945 |
| ETTR3 | 0.0058 | 0.0092 | 0.1832 | 0.0000 | 0.0022 | 0.0000 | 0.0061 | 1.0000 | 1.0000 | 1.0000 | 0.1801 | 1.0000 |
| ETSU | 0.0010 | 0.0002 | 0.0003 | 0.0003 | 0.0000 | 0.0000 | 0.0562 | 0.0004 | 0.1281 | 0.0000 | 0.2561 | 0.6209 |
| ETSAN | 0.3786 | 0.8455 | 0.0005 | 0.0007 | 0.0000 | 0.3372 | 0.1311 | 0.1205 | 0.0049 | 0.0000 | 0.5127 | 1.0000 |
| ETMA | 0.0550 | 1.0000 | 0.4831 | 0.3177 | 0.1334 | 0.0020 | 0.0000 | 0.6304 | 1.0000 | 0.0000 | 0.0000 | 1.0000 |
| ETBAT | 0.2399 | 0.5939 | 1.0000 | 0.7364 | 1.0000 | 1.0000 | 1.0000 | 1.0000 | 0.2145 | - | 0.0000 | 0.3849 |
| ETFA | 0.1046 | 0.4056 | 0.4687 | 0.0562 | 1.0000 | 0.0050 | 0.7188 | 0.0002 | 0.2209 | 0.0001 | 0.0002 | - |
| ETV | 0.0444 | 0.1743 | 0.0238 | 1.0000 | 0.5736 | 0.5736 | 1.0000 | 0.4785 | 0.4991 | 0.2092 | 1.0000 | - |
| IND | 0.4649 | 0.0000 | 0.0000 | 0.0009 | 0.0206 | 0.0008 | 0.0007 | 0.1112 | 0.6283 | 0.0000 | 0.0005 | - |
| MZB | 0.1073 | 0.0058 | 0.0015 | 1.0000 | 0.0020 | 0.0002 | 0.0256 | 0.9225 | 0.0010 | 0.0000 | 0.0020 | 0.0071 |
| MZD | 0.4341 | 0.5225 | 0.7748 | 1.0000 | 0.0000 | 0.0000 | 0.0002 | 0.0000 | 0.0001 | 0.0000 | 0.0000 | 0.0087 |
