# Supplementary material for: East Timor as an important source of cashew (Anacardium occidentale L.) genetic diversity
Source: PeerJ. 2023 Apr 24;11:e14894. doi: 10.7717/peerj.14894 (PMC10135414; doi:10.7717/peerj.14894)
Supplement: Table S2 — Statistical significance was assessed by running 10,000 iterations Monte Carlo Markov Chain (MCMC) test. p-values were corrected by multiple comparisons applying a sequential Bonferroni correction (p < 0.000298, [0.05/168]), p < 0.05, light blue; p < 0.000298, dark blue if present). [file peerj-11-14894-s002.docx]

**Supplementary Table S2.** Linkage Disequilibrium (LD) test for each locus-population combination using GenePop v4.5. Statistical significance was assessed by running 10,000 iterations Monte Carlo Markov Chain (MCMC) test. *p*-values were corrected by multiple comparisons applying a sequential Bonferroni correction (*p*<0.000298, [0.05/168]), *p*<0.05, light blue)

| **Locus Pair** | ***df*** | ***p-value*** |
| --- | --- | --- |
| mAor48 & mAoR6 | 47.753946 | 0.011389 |
| mAor48 & mAoR17 | 30.395375 | 0.344571 |
| mAoR6 & mAoR17 | 27.613632 | 0.485056 |
| mAor48 & mAoR7 | 32.837151 | 0.241793 |
| mAoR6 & mAoR7 | 42.855888 | 0.035938 |
| mAoR17 & mAoR7 | 36.194097 | 0.137722 |
| mAor48 & mAoR11 | 19.960269 | 0.865909 |
| mAoR6 & mAoR11 | 22.568514 | 0.754318 |
| mAoR17 & mAoR11 | 26.430276 | 0.549406 |
| mAoR7 & mAoR11 | 22.260554 | 0.769088 |
| mAor48 & mAoR3 | 28.416226 | 0.442563 |
| mAoR6 & mAoR3 | 23.479344 | 0.708695 |
| mAoR17 & mAoR3 | 25.653320 | 0.592094 |
| mAoR7 & mAoR3 | 26.987640 | 0.518920 |
| mAoR11 & mAoR3 | 29.444483 | 0.390251 |
| mAor48 & mAoR42 | 24.514105 | 0.654133 |
| mAoR6 & mAoR42 | 13.821288 | 0.988409 |
| mAoR17 & mAoR42 | 25.575726 | 0.596353 |
| mAoR7 & mAoR42 | 25.166468 | 0.618760 |
| mAoR11 & mAoR42 | 33.247229 | 0.226760 |
| mAoR3 & mAoR42 | 29.977132 | 0.364312 |
| mAor48 & mAoR52 | 27.472741 | 0.492632 |
| mAoR6 & mAoR52 | 25.104982 | 0.622115 |
| mAoR17 & mAoR52 | 25.003626 | 0.627638 |
| mAoR7 & mAoR52 | 32.246857 | 0.264582 |
| mAoR11 & mAoR52 | 22.887511 | 0.738649 |
| mAoR3 & mAoR52 | 29.427672 | 0.391083 |
| mAoR42 & mAoR52 | 27.725475 | 0.479064 |
| mAor48 & mAoR2 | 36.299907 | 0.135119 |
| mAoR6 & mAoR2 | 23.182966 | 0.723830 |
| mAoR17 & mAoR2 | 21.754908 | 0.792510 |
| mAoR7 & mAoR2 | 12.780735 | 0.993826 |
| mAoR11 & mAoR2 | 20.240266 | 0.855549 |
| mAoR3 & mAoR2 | 20.419426 | 0.848699 |
| mAoR42 & mAoR2 | 21.422293 | 0.807308 |
| mAoR52 & mAoR2 | 16.194895 | 0.962844 |
| mAor48 & mAoR33 | 18.997890 | 0.898201 |
| mAoR6 & mAoR33 | 14.587969 | 0.982464 |
| mAoR17 & mAoR33 | 23.038715 | 0.731100 |
| mAoR7 & mAoR33 | 31.470614 | 0.296584 |
| mAoR11 & mAoR33 | 30.364833 | 0.345993 |
| mAoR3 & mAoR33 | 24.423173 | 0.659015 |
| mAoR42 & mAoR33 | 11.101639 | 0.998169 |
| mAoR52 & mAoR33 | 28.523800 | 0.436968 |
| mAoR2 & mAoR33 | 18.967550 | 0.899134 |
| mAor48 & mAoR35 | 16.267385 | 0.878184 |
| mAoR6 & mAoR35 | 16.556237 | 0.866961 |
| mAoR17 & mAoR35 | 17.833742 | 0.810998 |
| mAoR7 & mAoR35 | 13.695165 | 0.953238 |
| mAoR11 & mAoR35 | 31.809684 | 0.131783 |
| mAoR3 & mAoR35 | 16.149084 | 0.882620 |
| mAoR42 & mAoR35 | 12.323318 | 0.976015 |
| mAoR52 & mAoR35 | 26.325170 | 0.336875 |
| mAoR2 & mAoR35 | 10.088544 | 0.994172 |
| mAoR33 & mAoR35 | 29.873411 | 0.188983 |
| mAor48 & mAoR47 | 34.691173 | 0.118491 |
| mAoR6 & mAoR47 | 24.990598 | 0.519507 |
| mAoR17 & mAoR47 | 17.431556 | 0.895466 |
| mAoR7 & mAoR47 | 15.143511 | 0.954653 |
| mAoR11 & mAoR47 | 18.083608 | 0.872710 |
| mAoR3 & mAoR47 | 25.328033 | 0.500473 |
| mAoR42 & mAoR47 | 17.207550 | 0.902679 |
| mAoR52 & mAoR47 | 12.279473 | 0.989485 |
| mAoR2 & mAoR47 | 12.737285 | 0.986191 |
| mAoR33 & mAoR47 | 14.567787 | 0.964735 |
| mAoR35 & mAoR47 | 28.195238 | 0.169301 |
| mAor48 & mAoR12 | 19.650661 | 0.807816 |
| mAoR6 & mAoR12 | 11.867362 | 0.991895 |
| mAoR17 & mAoR12 | 22.911876 | 0.637928 |
| mAoR7 & mAoR12 | 26.651311 | 0.427791 |
| mAoR11 & mAoR12 | 15.121565 | 0.955070 |
| mAoR3 & mAoR12 | 12.697200 | 0.986508 |
| mAoR42 & mAoR12 | 23.078731 | 0.628489 |
| mAoR52 & mAoR12 | 16.431958 | 0.925243 |
| mAoR2 & mAoR12 | 32.510894 | 0.176765 |
| mAoR33 & mAoR12 | 23.610331 | 0.598236 |
| mAoR35 & mAoR12 | 14.572010 | 0.879942 |
| mAoR47 & mAoR12 | 37.854463 | 0.062471 |
| mAor48 & mAoR16 | 17.405624 | 0.830854 |
| mAoR6 & mAoR16 | 35.091286 | 0.067059 |
| mAoR17 & mAoR16 | 20.067804 | 0.692914 |
| mAoR7 & mAoR16 | 27.664122 | 0.274461 |
| mAoR11 & mAoR16 | 24.815915 | 0.415819 |
| mAoR3 & mAoR16 | 24.807142 | 0.416301 |
| mAoR42 & mAoR16 | 26.197472 | 0.343222 |
| mAoR52 & mAoR16 | 18.345651 | 0.785926 |
| mAoR2 & mAoR16 | 26.260025 | 0.340104 |
| mAoR33 & mAoR16 | 11.798694 | 0.982082 |
| mAoR35 & mAoR16 | 9.001179 | 0.982894 |
| mAoR47 & mAoR16 | 33.745182 | 0.089301 |
| mAoR12 & mAoR16 | 19.485663 | 0.725617 |
| mAor48 & mAoR29 | 30.996760 | 0.317224 |
| mAoR6 & mAoR29 | 16.526198 | 0.957370 |
| mAoR17 & mAoR29 | 14.726030 | 0.981184 |
| mAoR7 & mAoR29 | 20.025788 | 0.863522 |
| mAoR11 & mAoR29 | 30.248131 | 0.351456 |
| mAoR3 & mAoR29 | 18.591667 | 0.910260 |
| mAoR42 & mAoR29 | 23.748966 | 0.694714 |
| mAoR52 & mAoR29 | 20.772781 | 0.834693 |
| mAoR2 & mAoR29 | 23.574823 | 0.703766 |
| mAoR33 & mAoR29 | 25.259175 | 0.613695 |
| mAoR35 & mAoR29 | 15.407333 | 0.908272 |
| mAoR47 & mAoR29 | 14.241614 | 0.969678 |
| mAoR12 & mAoR29 | 23.965868 | 0.577917 |
| mAoR16 & mAoR29 | 20.680152 | 0.540573 |
| mAor48 & mAoR41 | 22.589534 | 0.753296 |
| mAoR6 & mAoR41 | 14.357026 | 0.984456 |
| mAoR17 & mAoR41 | 25.480999 | 0.601549 |
| mAoR7 & mAoR41 | 20.757917 | 0.835295 |
| mAoR11 & mAoR41 | 15.890144 | 0.967418 |
| mAoR3 & mAoR41 | 29.042100 | 0.410395 |
| mAoR42 & mAoR41 | 27.236982 | 0.505372 |
| mAoR52 & mAoR41 | 42.837978 | 0.036081 |
| mAoR2 & mAoR41 | 13.392991 | 0.990966 |
| mAoR33 & mAoR41 | 21.054127 | 0.823085 |
| mAoR35 & mAoR41 | 11.630251 | 0.983762 |
| mAoR47 & mAoR41 | 26.020171 | 0.461996 |
| mAoR12 & mAoR41 | 21.145273 | 0.734427 |
| mAoR16 & mAoR41 | 15.251420 | 0.913186 |
| mAoR29 & mAoR41 | 16.187380 | 0.962963 |
