# Supplementary material for: East Timor as an important source of cashew (Anacardium occidentale L.) genetic diversity
Source: PeerJ. 2023 Apr 24;11:e14894. doi: 10.7717/peerj.14894 (PMC10135414; doi:10.7717/peerj.14894)
Supplement: Table S3 [file peerj-11-14894-s003.docx]

**Supplementary Table S3.** Pairwise *F*_ST_ (lower-left matrix) and *F*_ST_ ^ENA^ (upper-right matrix) between all populations in East Timor, Indonesia and Mozambique.

|  | ETK | ETNA | ETTR1 | ETTR2 | ETTR3 | ETSU | ETSAN | ETMA | ETBAT | ETFA | ETV | IND | MZB | MZD |
| --- | --- | --- | --- | --- | --- | --- | --- | --- | --- | --- | --- | --- | --- | --- |
| ETK | **0** | 0.2248 | 0.3292 | 0.3860 | 0.2020 | 0.3227 | 0.3345 | 0.3347 | 0.4090 | 0.2763 | 0.2868 | 0.2688 | 0.4002 | 0.4105 |
| ETNA | 0.4217 | **0** | 0.4881 | 0.4299 | 0.2750 | 0.3610 | 0.3157 | 0.1703 | 0.3051 | 0.2575 | 0.2967 | 0.3272 | 0.5102 | 0.4056 |
| ETTR1 | 0.4883 | 0.5494 | **0** | 0.2505 | 0.2226 | 0.4404 | 0.4267 | 0.4512 | 0.4707 | 0.3636 | 0.3492 | 0.2846 | 0.5987 | 0.5572 |
| ETTR2 | 0.5266 | 0.5433 | 0.4261 | **0** | 0.1666 | 0.5057 | 0.4263 | 0.4091 | 0.3270 | 0.3651 | 0.3424 | 0.2646 | 0.6222 | 0.4881 |
| ETTR3 | 0.4361 | 0.4491 | 0.4187 | 0.3959 | **0** | 0.2815 | 0.1990 | 0.2019 | 0.1885 | 0.1508 | 0.1632 | 0.1255 | 0.4661 | 0.3074 |
| ETSU | 0.5287 | 0.5020 | 0.5254 | 0.5367 | 0.4567 | **0** | 0.3121 | 0.3227 | 0.4463 | 0.3199 | 0.4693 | 0.3057 | 0.4670 | 0.4986 |
| ETSAN | 0.4715 | 0.4308 | 0.5346 | 0.5156 | 0.3880 | 0.4796 | **0** | 0.1546 | 0.2014 | 0.2408 | 0.3235 | 0.2865 | 0.4933 | 0.3976 |
| ETMA | 0.4542 | 0.3119 | 0.5115 | 0.5214 | 0.4040 | 0.4729 | 0.3095 | **0** | 0.1179 | 0.1475 | 0.3169 | 0.2706 | 0.5080 | 0.2717 |
| ETBAT | 0.5008 | 0.3758 | 0.5356 | 0.4882 | 0.4049 | 0.5101 | 0.3742 | 0.2785 | **0** | 0.1929 | 0.3468 | 0.3327 | 0.5679 | 0.2760 |
| ETFA | 0.4489 | 0.3891 | 0.5268 | 0.5214 | 0.3932 | 0.4706 | 0.3826 | 0.3009 | 0.3622 | **0** | 0.2396 | 0.1785 | 0.6150 | 0.2866 |
| ETV | 0.4777 | 0.4316 | 0.5148 | 0.4932 | 0.3860 | 0.5528 | 0.4477 | 0.4189 | 0.4364 | 0.4125 | **0** | 0.1942 | 0.4483 | 0.3842 |
| IND | 0.4901 | 0.5001 | 0.4633 | 0.4554 | 0.3660 | 0.4653 | 0.4677 | 0.4511 | 0.4826 | 0.3917 | 0.4109 | **0** | 0.5398 | 0.4064 |
| MZB | 0.5206 | 0.5408 | 0.5983 | 0.5982 | 0.5526 | 0.5372 | 0.5241 | 0.5064 | 0.5356 | 0.5354 | 0.5646 | 0.5892 | **0** | 0.3847 |
| MZD | 0.5147 | 0.5056 | 0.5875 | 0.5805 | 0.5172 | 0.5744 | 0.5125 | 0.4393 | 0.4353 | 0.4573 | 0.5120 | 0.5390 | 0.4710 | **0** |
