# Supplementary material for: East Timor as an important source of cashew (Anacardium occidentale L.) genetic diversity
Source: PeerJ. 2023 Apr 24;11:e14894. doi: 10.7717/peerj.14894 (PMC10135414; doi:10.7717/peerj.14894)
Supplement: Figure S2 — Legend: Manatuto, Bobonaro, Baucau, Covalima, Manufahi, Viqueque, and Indonesia) [file peerj-11-14894-s006.pdf]

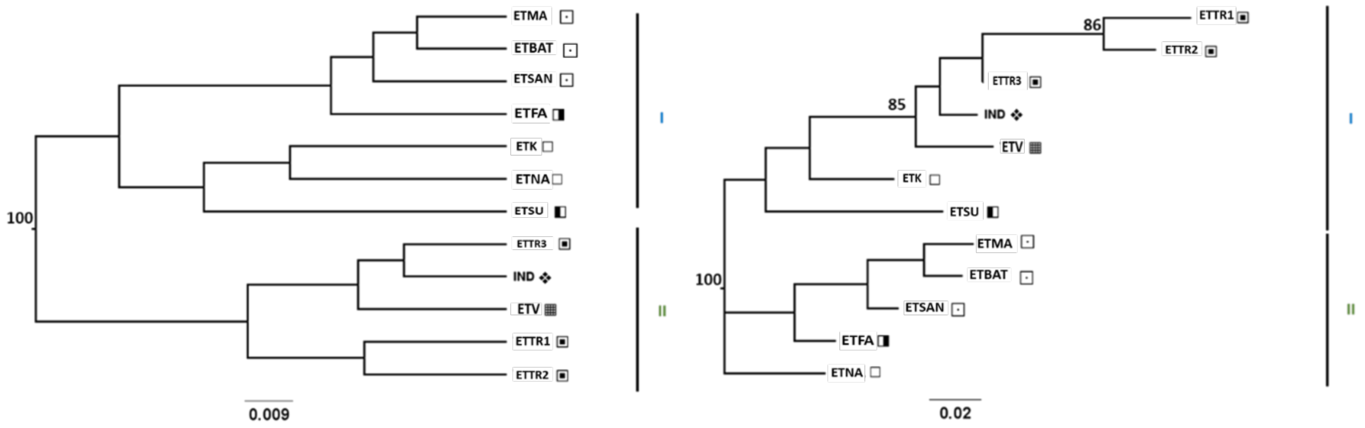

Supplementary Figure S2. UPGMA (**A**) and NJ (**B**) trees generated using matrix Nei's *D* distance, respectively, representing the cashew populations of East Timor and Indonesia.

Legend: □ Manatuto, ⊡ Bobonaro, ▣ Baucau, ◧ Covalima, ◨ Manufahi, ▦ Viqueque, and ❖ Indonesia.

Formatou: Tipo de letra: Negrito, Inglês (Estados Unidos)

Formatou: Tipo de letra: Negrito

Eliminou: from

Eliminou: -

Eliminou: ,

Eliminou: (

Eliminou:)
