# Supplementary material for: East Timor as an important source of cashew (Anacardium occidentale L.) genetic diversity
Source: PeerJ. 2023 Apr 24;11:e14894. doi: 10.7717/peerj.14894 (PMC10135414; doi:10.7717/peerj.14894)
Supplement: Figure S3 — STRUCTURE ad hoc statistics retrieved by Structure Harvester using 1 to 15 possible clusters (K). Variation of ΔK values according to the method outlined by Evanno, Regnaut & Goudet (2005) for populations from East Timor, Indonesia, and Mozambique. [file peerj-11-14894-s007.pdf]

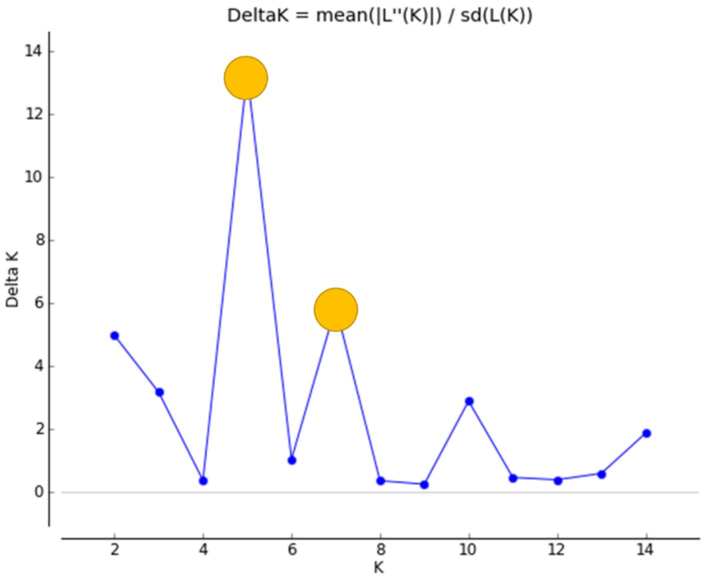

Supplementary Figure S3. STRUCTURE ad hoc statistics retrieved by Structure Harvester using 1 to 15 possible clusters (*K*). Variation of ΔK values according to the method outlined by Evanno et al. (2005) for populations from East Timor, Indonesia and Mozambique.

Eliminou: [41]

Eliminou: method

Eliminou: -
