# Supplementary material for: East Timor as an important source of cashew (Anacardium occidentale L.) genetic diversity
Source: PeerJ. 2023 Apr 24;11:e14894. doi: 10.7717/peerj.14894 (PMC10135414; doi:10.7717/peerj.14894)
Supplement: Figure S4 — A K value of 10 (the lowest BIC value) represents the best summary of the data for the populations of East Timor, Indonesia, and Mozambique. [file peerj-11-14894-s008.pdf]

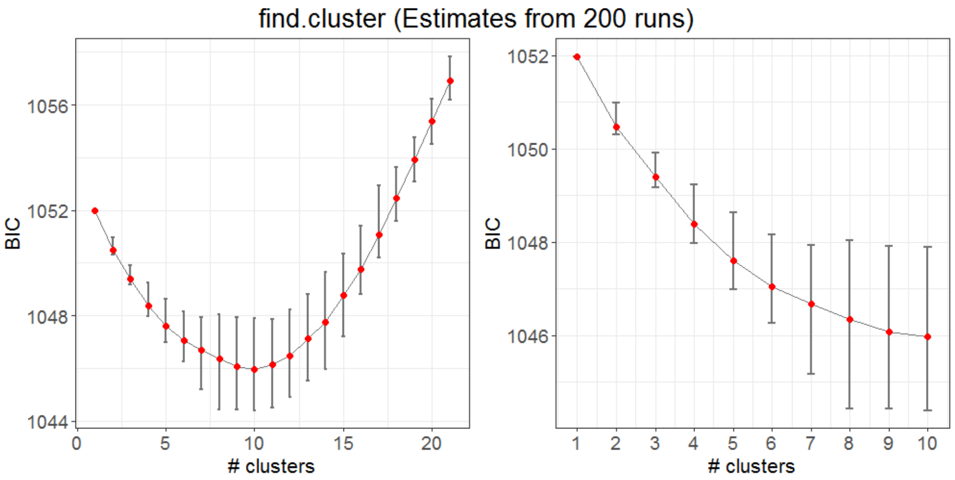

Supplementary Figure S4. DAPC results inference of the number of clusters using the *find.clusters* function with a *K* = 10 (left) and *K* = 5 (right). A *K* value of 10 (the lowest BIC value) represents the best summary of the data for the populations of East Timor, Indonesia, and Mozambique.

Eliminou:

Eliminou: -
