# Supplementary material for: East Timor as an important source of cashew (Anacardium occidentale L.) genetic diversity
Source: PeerJ. 2023 Apr 24;11:e14894. doi: 10.7717/peerj.14894 (PMC10135414; doi:10.7717/peerj.14894)
Supplement: Figure S5 — Scatterplot shows the two principal components of the DAPC, and clusters are numbered and displayed by different colors, while dots represent individuals. The 2 Discriminant Functions hereby represented explain 93% of cumulative variance of the dataset. [file peerj-11-14894-s009.pdf]

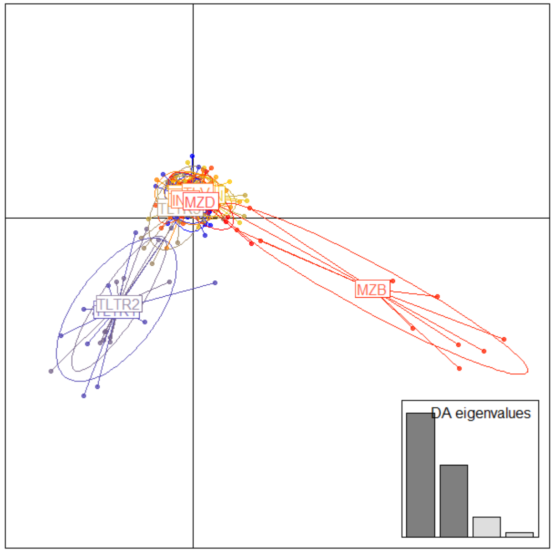


**Supplementary Figure S5.** Scatterplot of the DAPC analysis of $K = 5$ assignment. Scatterplot shows the two principal components of the DAPC, and clusters are numbered and displayed by different colors, while dots represent individuals. The 2 Discriminant Functions hereby represented explain 93% of cumulative variance of the dataset

**Eliminou:** for
