# Supplementary material for: East Timor as an important source of cashew (Anacardium occidentale L.) genetic diversity
Source: PeerJ. 2023 Apr 24;11:e14894. doi: 10.7717/peerj.14894 (PMC10135414; doi:10.7717/peerj.14894)
Supplement: Figure S6 — (A) Allele loading plots from DF1 and (B) from DF2, after assigning 0.045 as the threshold. [file peerj-11-14894-s010.pdf]

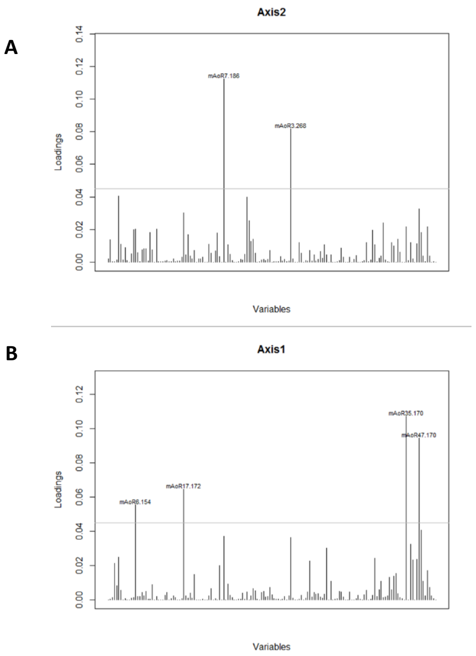

Supplementary Figure S6. Loading plots of the two discriminant functions following the DAPC analysis with a *K* = 5. (**A**) Allele loading plots from DF1 and (**B**) from DF2, after assigning a 0.045 as threshold.

Eliminou: D

Eliminou: F

Formatou: Tipo de letra: Negrito

Formatou: Tipo de letra: Negrito

Formatou: Inglês (Estados Unidos)
