# Supplementary material for: East Timor as an important source of cashew (Anacardium occidentale L.) genetic diversity
Source: PeerJ. 2023 Apr 24;11:e14894. doi: 10.7717/peerj.14894 (PMC10135414; doi:10.7717/peerj.14894)
Supplement: Figure S7 — Variation of ΔK values according to the methods outlined by Evanno, Regnaut & Goudet (2005) for populations from East Timor and Indonesia. [file peerj-11-14894-s011.pdf]

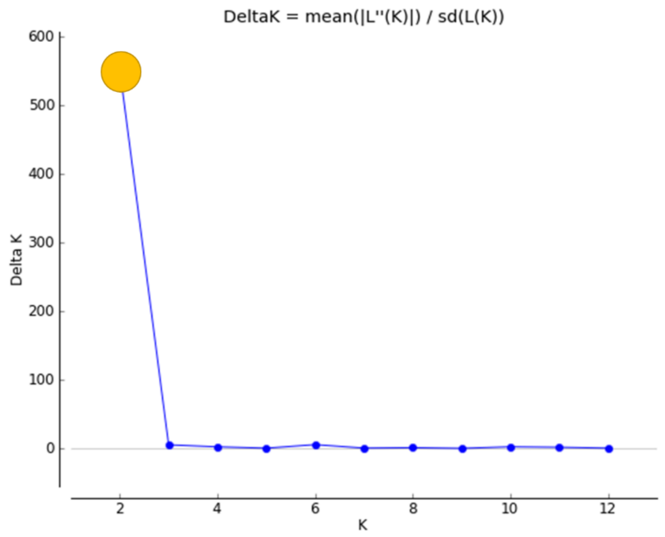

Supplementary Figure S7. STRUCTURE ad hoc statistics retrieved by Structure Harvester using 1 to 12 possible clusters (*K*). Variation of ΔK values according to the method outlined by Evanno *et al.* (2005) for populations from East Timor and Indonesia.

Eliminou: method

Eliminou: -
