# Supplementary material for: East Timor as an important source of cashew (Anacardium occidentale L.) genetic diversity
Source: PeerJ. 2023 Apr 24;11:e14894. doi: 10.7717/peerj.14894 (PMC10135414; doi:10.7717/peerj.14894)
Supplement: Figure S8 [file peerj-11-14894-s012.pdf]

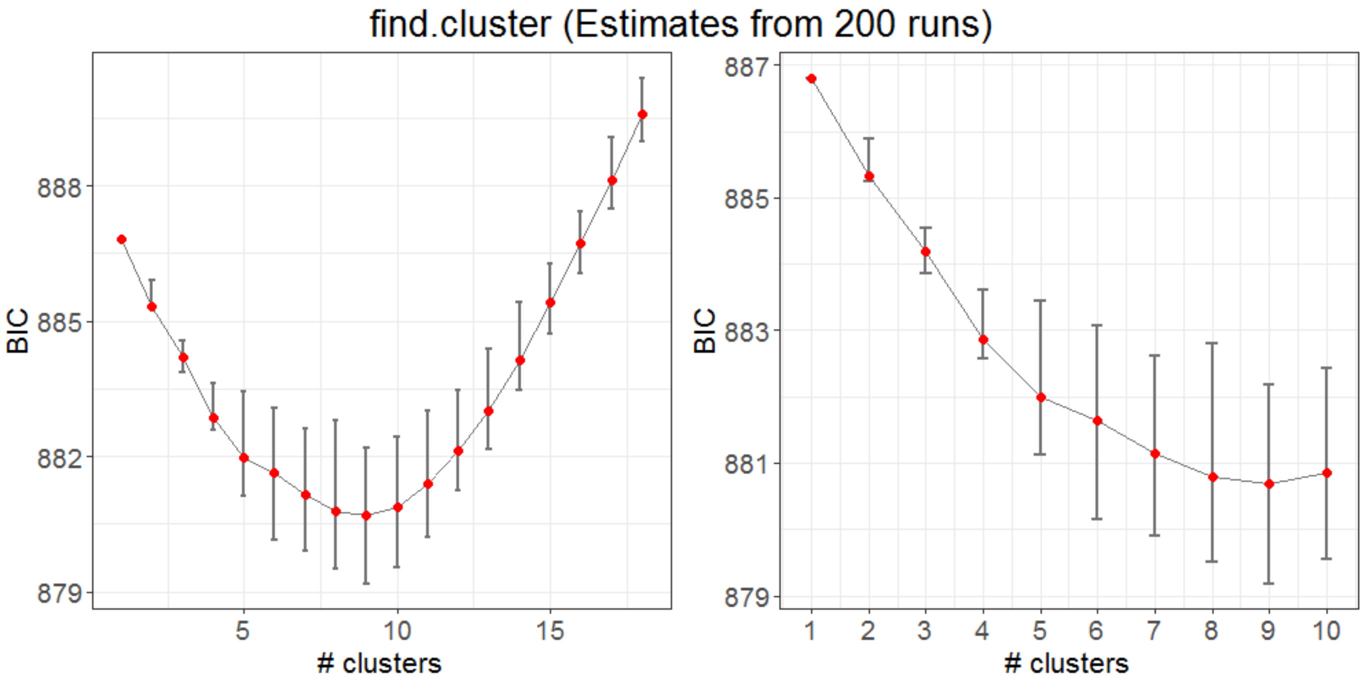

Supplementary Figure S8. Number of clusters inferred by the DAPC find.*clusters* function with a *K* = 5 and *K* = 2.
