# Supplementary material for: East Timor as an important source of cashew (Anacardium occidentale L.) genetic diversity
Source: PeerJ. 2023 Apr 24;11:e14894. doi: 10.7717/peerj.14894 (PMC10135414; doi:10.7717/peerj.14894)
Supplement: Figure S9 [file peerj-11-14894-s013.pdf]

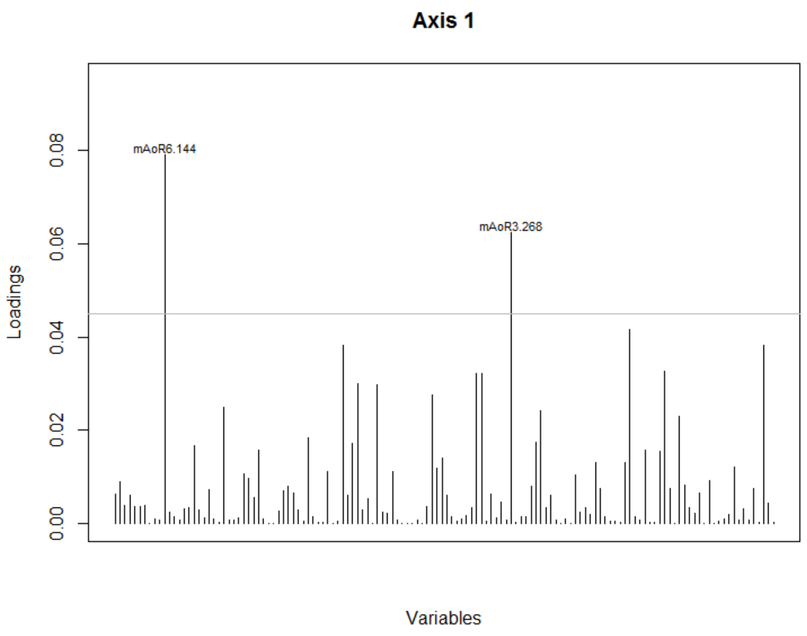

Supplementary Figure S9. Loading plot of the DF1 following the DAPC analysis with a $K = 2$, after assigning 0.045 as the threshold.

Eliminou: a
